# A Mimicking-of-DNA-Methylation-Patterns Pipeline for Overcoming the Restriction Barrier of Bacteria

**DOI:** 10.1371/journal.pgen.1002987

**Published:** 2012-09-27

**Authors:** Guoqiang Zhang, Wenzhao Wang, Aihua Deng, Zhaopeng Sun, Yun Zhang, Yong Liang, Yongsheng Che, Tingyi Wen

**Affiliations:** 1 Department of Industrial Microbiology and Biotechnology, Institute of Microbiology, Chinese Academy of Sciences, Beijing, China; 2 State Key Laboratory of Mycology, Institute of Microbiology, Chinese Academy of Sciences, Beijing, China; 3 Department of Natural Products Chemistry, Beijing Institute of Pharmacology and Toxicology, Beijing, China; Universidad de Sevilla, Spain

## Abstract

Genetic transformation of bacteria harboring multiple Restriction-Modification (R-M) systems is often difficult using conventional methods. Here, we describe a mimicking-of-DNA-methylation-patterns (MoDMP) pipeline to address this problem in three difficult-to-transform bacterial strains. Twenty-four putative DNA methyltransferases (MTases) from these difficult-to-transform strains were cloned and expressed in an *Escherichia coli* strain lacking all of the known R-M systems and orphan MTases. Thirteen of these MTases exhibited DNA modification activity in Southwestern dot blot or Liquid Chromatography–Mass Spectrometry (LC–MS) assays. The active MTase genes were assembled into three operons using the *Saccharomyces cerevisiae* DNA assembler and were co-expressed in the *E. coli* strain lacking known R-M systems and orphan MTases. Thereafter, results from the dot blot and restriction enzyme digestion assays indicated that the DNA methylation patterns of the difficult-to-transform strains are mimicked in these *E. coli* hosts. The transformation of the Gram-positive *Bacillus amyloliquefaciens* TA208 and *B. cereus* ATCC 10987 strains with the shuttle plasmids prepared from MoDMP hosts showed increased efficiencies (up to four orders of magnitude) compared to those using the plasmids prepared from the *E. coli* strain lacking known R-M systems and orphan MTases or its parental strain. Additionally, the gene coding for uracil phosphoribosyltransferase (*upp*) was directly inactivated using non-replicative plasmids prepared from the MoDMP host in *B. amyloliquefaciens* TA208. Moreover, the Gram-negative chemoautotrophic *Nitrobacter hamburgensis* strain X14 was transformed and expressed Green Fluorescent Protein (GFP). Finally, the sequence specificities of active MTases were identified by restriction enzyme digestion, making the MoDMP system potentially useful for other strains. The effectiveness of the MoDMP pipeline in different bacterial groups suggests a universal potential. This pipeline could facilitate the functional genomics of the strains that are difficult to transform.

## Author Summary

Approximately 95% of the genome-sequenced bacteria harbor Restriction-Modification (R-M) systems. R-M systems usually occur in pairs, *i.e.*, DNA methyltransferases (MTases) and restriction endonucleases (REases). REases can degrade invading DNA to protect the cell from infection by phages. This protecting machinery has also become the barrier for experimental genetic manipulation, because the newly introduced DNA would be degraded by the REases of the transformed bacteria. In this study we have developed a pipeline to protect DNA by methylation from cleavage by host REases. Multiple DNA MTases were cloned from three difficult-to-transform bacterial strains and co-expressed in an *E. coli* strain lacking all of the known endogenous R-M systems and orphan MTases. Thus, the DNA methylation patterns of these strains have become similar to that of the difficult-to-transform strains. Ultimately, the DNA prepared from these *E. coli* strains can overcome the R-M barrier of the bacterial strains that are difficult to transform and achieve genetic manipulation. The effectiveness of this pipeline in different bacterial groups suggests a universal potential. This pipeline could facilitate functional genomics of bacterial strains that are difficult to transform.

## Introduction

Experimental genetic manipulation has been an essential tool for gaining insight into the significance of bacterial metabolism, physiology and pathogenesis [1], [2] and has been instrumental in developing microbial biotechnology [3]. To date, only a limited proportion of the laboratory culturable bacterial species are amenable to genetic manipulation. Among these manipulation-friendly species, many strains are refractory to transformation by exogenous DNA. The currently available laboratory model species satisfied the research need for genetic uniformity, but the handicap in genetic manipulation is a challenge when exploring the unique traits of these non-model species/strains [4].

Restriction-Modification (R-M) systems are composed of restriction enzymes (REases) and DNA methyltransferases (MTases). These systems are widespread in both bacteria and archaea. Approximately 95% of the genome-sequenced bacteria harbor R-M systems, and 33% carry more than four REases [5]. R-M systems have been classified into four groups depending on their subunit composition, cleavage sites, sequence specificity and cofactor requirement [6]. Type I, II and III REases cleave unmethylated DNA at specific sites, and Type IV cut methylated DNA with foreign patterns [6]. R-M systems are believed to act as defenses to protect the prokaryotic cells against invading DNA; exogenous DNA with foreign methylation patterns are recognized and rapidly degraded [7]. Inevitably, this defensive machinery hinders the experimental genetic manipulation of many bacteria species. Moreover, genetic modification becomes even more difficult when the targeted bacteria carry multiple R-M systems.

The *Nitrobacter hamburgensis* X14 strain oxidizes nitrite to conserve energy and is commonly used in nitrification research [8]. Although the strain was isolated more than 100 years ago [9], limited research on this strain has been published due to the lack of genetic manipulation tools. Genomic sequencing has revealed eleven sets of R-M genes in *N. hamburgensis* X14 [10]. *Bacillus cereus* ATCC 10987 is a non-lethal strain in the same genetic subgroup as *B. anthracis* [11]. Although genetic manipulation has been routine in many other *B. cereus* strains [12], limited research has been performed in *B. cereus* ATCC 10987 due to its resistance to genetic manipulation. Transformation of *B. cereus* ATCC 10987 has been performed with DNA prepared from *Bacillus subtilis* with low efficiency [13], [14], and four REases have recently been characterized [15]. *Bacillus amyloliquefaciens* TA208 is an industrial guanosine-producing strain [16] and has been reported to be transformed at low efficiencies with plasmids prepared from *Escherichia coli* [17].

Here, we describe a mimicking-of-DNA-methylation-patterns (MoDMP) pipeline. An *E. coli* strain lacking all of the known six characterized R-M systems and orphan MTases was generated to prevent unintentional modification of propagated plasmids or cleavage of DNA with foreign methylation patterns. After expressing multiple active MTases from the target bacteria in the *E. coli* strain lacking known R-M systems and orphan MTases, the DNA methylation patterns of *E. coli* were altered to reflect the patterns of the target bacteria. Plasmids prepared from these hosts escaped the host REases, and genetic manipulation could be readily achieved. The pipeline was shown to be effective in all of the three aforementioned strains which are difficult to transform using conventional methods. We report the first genetic transformation of *Nitrobacter*, the improvement of transformation efficiency by exogenous plasmids in *B. cereus* ATCC 10987 and *B. amyloliquefaciens* TA208 using the MoDMP pipeline, and direct mutagenesis using non-replicative plasmids in *B. amyloliquefaciens* TA208. The MoDMP pipeline may be readily adapted to bacteria carrying multiple R-M systems.

## Results

### Generation and validation of the *E. coli* strain lacking known R-M systems and orphan MTases

To avoid the unintentional activation of the Type IV R-M systems in the target bacteria, plasmids that are to be used for genetic transformation should be prepared from an *E. coli* host that does not methylate DNA (*dam*- *dcm*- *hsdRMS*-). Moreover, the expression of MTases in *E. coli* would induce foreign patterns of modification on the *E. coli* chromosomal DNA; therefore, the REases that restrict methylated DNA (Mrr, McrA and MrcBC) should be inactivated in the MoDMP host. To date, three *E. coli* strains that do not methylate DNA or restrict DNA with foreign methylation patterns have been described (*E. coli* DB24 [18], *E. coli* HST04 from Clontech and *E. coli* JTU007 [19]). In this study, an *E. coli* mutant lacking all of the six characterized R-M systems and orphan MTases genes, namely strain EC135, was generated in the *E. coli* TOP10 background by deleting the *dam* and *dcm* genes. A wild-type *recA* allele was introduced into the strain prior to *dam* inactivation to counteract the inviability of the *dam recA* double mutant strain [20]. The construction of the *E. coli* EC135 strain is explained in detail in the Supporting Information Methods (Text S1), and validation of the strain is described in the Supporting Information Results (Text S1 and Figure S1).

### Cloning and characterization of putative MTases

MTases modify DNA by adding a methyl group to the individual bases, thereby preventing DNA cleavage by the corresponding REases. In the MoDMP procedure, MTases from the difficult-to-transform bacterial strains were used to protect DNA from being degraded by the REases. The genes of 24 putative MTases including two belonging to Type I R-M systems, 19 belonging to Type II R-M systems, two belonging to Type III R-M systems, and one orphan MTase were cloned from the genomes of the three difficult-to-transform strains into the pBAD43 vector (Table 1).

**Table 1:** The MTases involved in this study and their characteristics.

MTase	Type	Modification base	Sequence specificity
*B. amyloliquefaciens* TA208			
BAMTA208_6525	II	m4C	RGATCY, TGATCA (P)
BAMTA208_6715	orphan	m5C	GGCC, GCNGC, GDGCHC
BAMTA208_14440	II	ND	
BAMTA208_19835	II	m5C	GCWGC
BAMTA208_16660	II	m4C	GGATCC
			
*B. cereus* ATCC 10987			
BCE_0841-CE_0842	I	ND	
BCE_0839-CE_0842	I	ND	
BCE_0365	II	m5C	GCWGC
BCE_0392	II	m6A^a^	
BCE_0393	II	m5C	GCWGC, GGCC, CCGG, GGNNCC, GCGCGC, GGWCC, CCWGG (P)
BCE_4605	II	m5C	GGWCC
BCE_5606	II	m4C	ACGGC(N)_12/14_
BCE_5607	II	m4C	ACGGC(N)_12/14_
BCE_1018	III	ND	
			
*N. hamburgensis* X14			
Nham_0569	II	m6A	GATC (P), CAGCTG (P), AATATT (P), ACTAGT (P), CAATTG (P), CATG (P), ATTAAT (P), GANTC (P)
Nham_0582	III	m6A^a^	
Nham_0803	II	ND^b^	
Nham_0842	II	ND	
Nham_1185	II	ND	
Nham_1353	II	ND	
Nham_2515	II	ND	
Nham_3225	II	m6A^a^	GANTC
Nham_3845	II	ND	
Nham_4499	II	ND	

a Weak signal in dot blot assays and verified by HPLC-QTOF/MS analysis;

b Weak m6A signal in dot blot assays and not detected in HPLC-QTOF/MS analysis.

Putative modification bases of the MTases have been underlined. R = A or G, Y = C or T, N = A or T or G or C, D = A or G or T, H = A or C or T, W = A or T. P, partial modification. ND, not detected.

**Figure 1:**
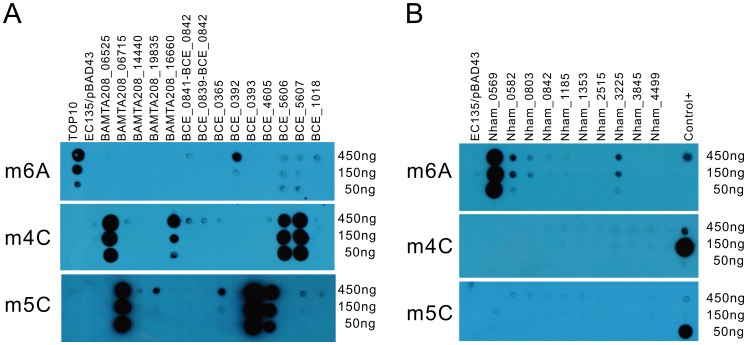
Dot blot assay for individual MTase activity. Serially diluted DNA (450 ng–150 ng) was used to test the *in vivo* methylation by MTases. (A) DNA methylated by the MTases from *B. amyloliquefaciens* TA208 and *B. cereus* ATCC 10987. (B) DNA methylated by the MTases from *N. hamburgensis* X14. Antibodies against m6A, m4C, and m5C were used in the upper, middle, and lower panels, respectively. DNA from the *E. coli* EC135 strain harboring pBAD43 was used as negative control. The identical DNA, 150 ng DNA of the *E. coli* TOP10 strain, 150 ng of *E. coli* EC135 DNA *in vivo* methylated by M.BamHI and 150 ng of *E. coli* EC135 DNA *in vivo* methylated by M.AluI (arranged from top to bottom), were used as controls in each “Control+” column for the m6A, m4C, and m5C experiments. All experiments were repeated at least three times, and representative results are shown.

To date, three types of methyl-transferring activity have been described for bacterial DNA MTases, namely *N*6-methyladenine (m6A), *N*4-methylcytosine (m4C) and 5-methylcytosine (m5C) modifications. Dot blot assays were conducted to detect the modified bases in the total genomic DNA of the *E. coli* EC135 strains expressing individual MTase using antibodies against m6A, m4C and m5C. In total, 13 of the putative MTase genes exhibited methyl transfer activity to DNA (Figure 1), and the bases they modified are summarized in Table 1. The spots of the dot blots were also scanned and quantified, and the relative intensity of each spot is shown in Figure S2.

**Figure 2:**
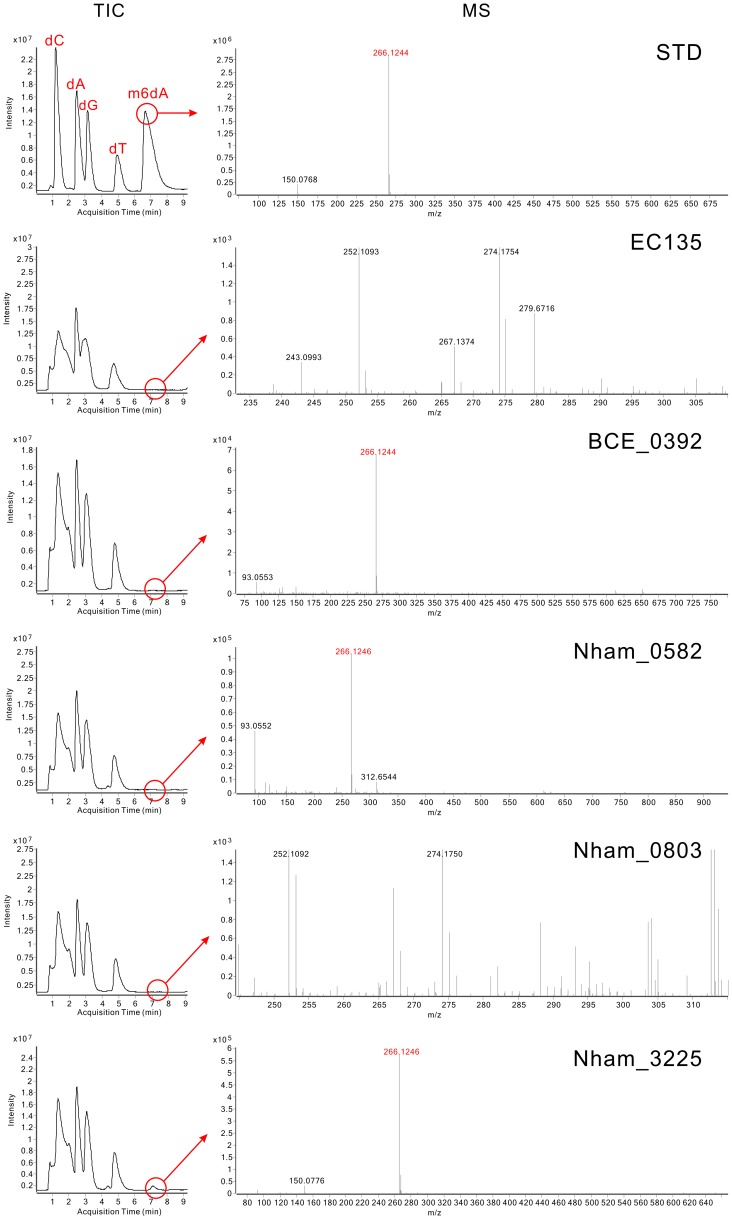
Analysis of m6dA in the DNA of MTases-expressing *E. coli* strains using HPLC-QTOF/MS. TIC plots represent the Total Ion Chromatograms of eluted components in the digested DNA samples, and MS plots represent the Mass Spectrum at the corresponding m6dA retention time. In the STD panel, 1.5 µg of dC (deoxycytidine), dA (deoxyadenosine), dG (deoxyguanosine), dT (deoxythymidine) and m6dA (N6-methyl-2′-deoxyadenosine) nucleoside standards were analyzed. The retention time of standard m6dA was 7.4 min, and the *m/z* was 266.12. In other panels, digested genomic DNA was used; the ion chromatogram was extracted at the retention time of m6dA and shown as MS plots.

The hybridization signals of BCE_0392, Nham_0582, Nham_0803 and Nham_3225 were weak in the dot blot experiments. To confirm their activity, the total DNA of the *E. coli* EC135 strains expressing these four MTases individually were digested to deoxynucleosides, and Liquid Chromatography-Mass Spectrometry (LC-MS) assays were performed to detect *N*6-methyl-2′-deoxyadenosine (m6dA) in the DNA. In High Performance Liquid Chromatography-Quadrupole Time-of-Flight/Mass Spectrometry (HPLC-QTOF/MS) analysis, m6dA (*m/z* 266.12) was readily detected in the digested DNA of the BCE_0392-, Nham_0582- and Nham_3225-expressing strains in the MS spectrum at the corresponding retention time of standard m6dA, validating that BCE_0392, Nham_0582, and Nham_3225 displayed DNA m6A modification activity in *E. coli* (Figure 2). Xu *et al*. has reported that MTase activity was not detected for *in vivo* translated BCE_0392 protein using [H^3^]AdoMet and phage *λ* DNA or pXbaI plasmid DNA as substrate [15]. This might either be caused by the mis-folding of *in vivo* translated BCE_0392 protein or by the absence of BCE_0392 recognition sites from the substrate DNA they used.

Although m6dA was not detected for Nham_0803 in the HPLC-QTOF/MS analysis (Figure 2), the MTase activity of Nham_0803 could not be ruled out, since the DNA of the Nham_0803-expressing strain displayed slight but noticeable signal increase compared with the *E. coli* EC135 strain harboring empty vector (Figure S2). Other more sensitive and targeted MS approaches could be useful in detecting the possible modified nucleoside conferred by Nham_0803 [21].

It is noteworthy that the *E. coli* strain EC135 expressing the Nham_0569 MTase grows much slower than the control strain or strains expressing other MTases (Nham_0803 and Nham_3225), and the final biomass of strain EC135 carrying Nham_0569 was about 60% of that for the control strains (Figure S3). This growth retardation in *E. coli* may be attributed to the toxicity of the Nham_0569 MTase. The *E. coli* EC135 strain lacks methylation-dependent REases activity, which will cleave its own DNA when foreign methylation patterns are detected, leading to cell death; however, the modification of m6A by the Nham_0569 MTase may occur on sequences overlapping with Dam sites in *E. coli*, which participates in DNA mismatch repair and replication initiation. Consequently, the premature and untimely methylation of DNA may interfere with strain proliferation [22]. Subsequent sequence specificity analysis has revealed that Nham_0569 modified GATC sequences (see below).

### Co-expression of multiple active MTases

**Figure 3:**
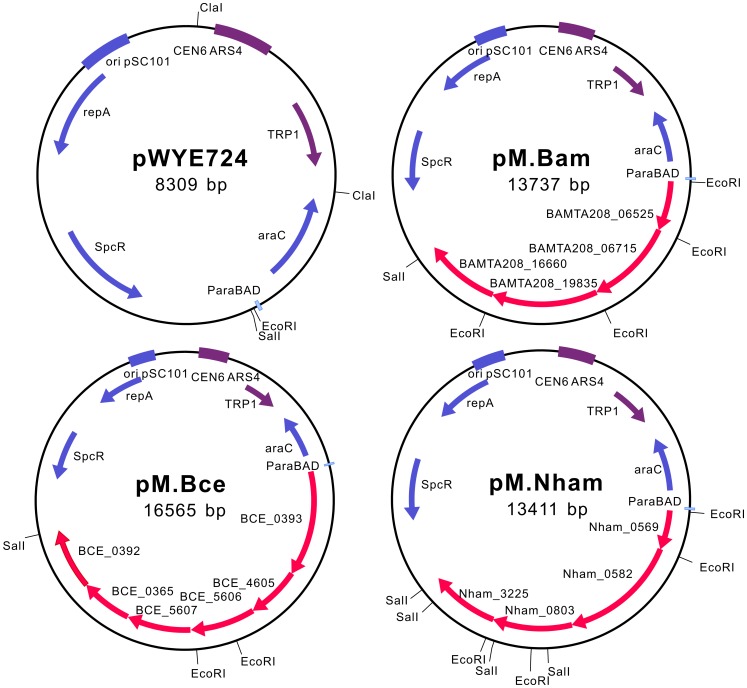
Schematic diagrams of pMoDMP plasmids. *S. cerevisiae* replication and screening elements are marked in purple; elements for replication, screening and protein expression in *E. coli* are marked in blue, and MTase genes are marked in red. pM.Bam, pM.Bce and pM.Nham carry MTase genes from *B. amyloliquefaciens* TA208, *B. cereus* ATCC 10987 and *N. hamburgensis* X14, respectively.

To mimic the DNA methylation patterns of the strains that are difficult to transform, we co-expressed the active MTases from each strain. By taking advantage of the high rates of recombination in *Saccharomyces cerevisiae*, MTase genes, with optimized ribosome binding site (RBS) for expression in *E. coli*, were inserted into the pWYE724 backbone to form three operons. The diagrams of the pMoDMP plasmids are shown in Figure 3. The insertion of the MTase genes was verified by multiple methods, including PCR analysis of plasmids, restriction digestion (Figure S4) and DNA sequencing. The protocol for DNA assembly in *S. cerevisiae* is very powerful, and up to eight MTase genes from *Nitrosococcus oceani* ATCC 19707 could be readily assembled in our lab (Zhang *et al.*, unpublished).

**Figure 4:**
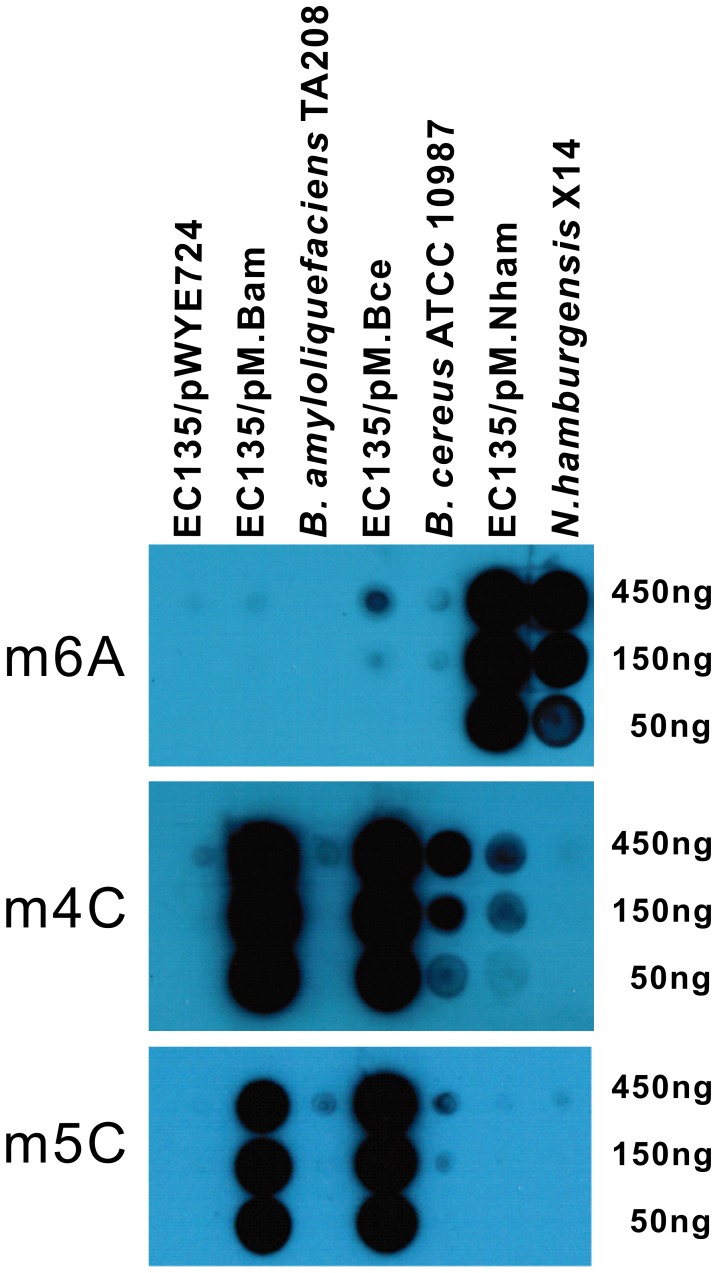
Dot blot assay for co-expression of multiple MTases. Serial dilutions of DNA (450 ng–50 ng) from the *E. coli* EC135 strain harboring pM.Bam, pM.Bce or pM.Nham were tested, and the corresponding DNA from the native host of the MTases was used as positive controls. DNA of the *E. coli* EC135 strain harboring pWYE724 was used as a negative control. Antibodies against m6A, m4C and m5C were used in the upper, middle, and lower panels, respectively. All experiments were repeated at least three times, and representative results are shown.

DNA from the *E. coli* EC135 strain expressing multiple MTases was also tested by dot blot assay, with the DNA of the parent strains as the positive controls (Figure 4). DNA from the co-expression strains exhibited multiple methylation signals, indicating the alteration of the DNA methylation patterns in *E. coli*. It is worth noting that the m4C and m5C signals in *B. amyloliquefaciens* strain TA208, the m5C signal in *B. cereus* strain ATCC 10987 and the m4C signal in *N. hamburgensis* strain X14 were much weaker when compared with their corresponding MTase over-expressing *E. coli* strain. This signal weakness could be attributed to the different number of MTase target sequences between the genomic sequences of *E. coli* and the parent strains or to the fact that the *B. amyloliquefaciens* TA208 strain is an adenine auxotroph, which limits the availability of *S*-adenosylmethionine (AdoMet). However, pMK4 plasmid DNA prepared from the TA208 strain is resistant to BamHI digestion, which is a homolog to the restriction subunit of the BAMTA208_16650-BAMTA208_16660 systems (see below). Thus, regulational expression of the R-M systems could also explain the weak blot signals in the parent strains; Hegna *et al.* has reported that the R-M system is activated when *B. cereus* is grown in the presence of exogenous DNA [23].

### The MoDMP pipeline increased the plasmid transformation efficiency of *Bacillus*

To determine the efficacy of the MoDMP pipeline, various shuttle plasmids carrying divergent replicons and conferring different antibiotic resistance were used to transform *B. amyloliquefaciens* TA208 and *B. cereus* ATCC 10987. Prior to transforming *Bacillus*, the shuttle plasmids were methylated *in vivo* when transformed into the *E. coli* EC135 strain harboring the pMoDMP plasmids. *B. amyloliquefaciens* TA208 and *B. cereus* ATCC 10987 were transformed by these plasmids, and the transformation efficiencies were calculated.

**Figure 5:**
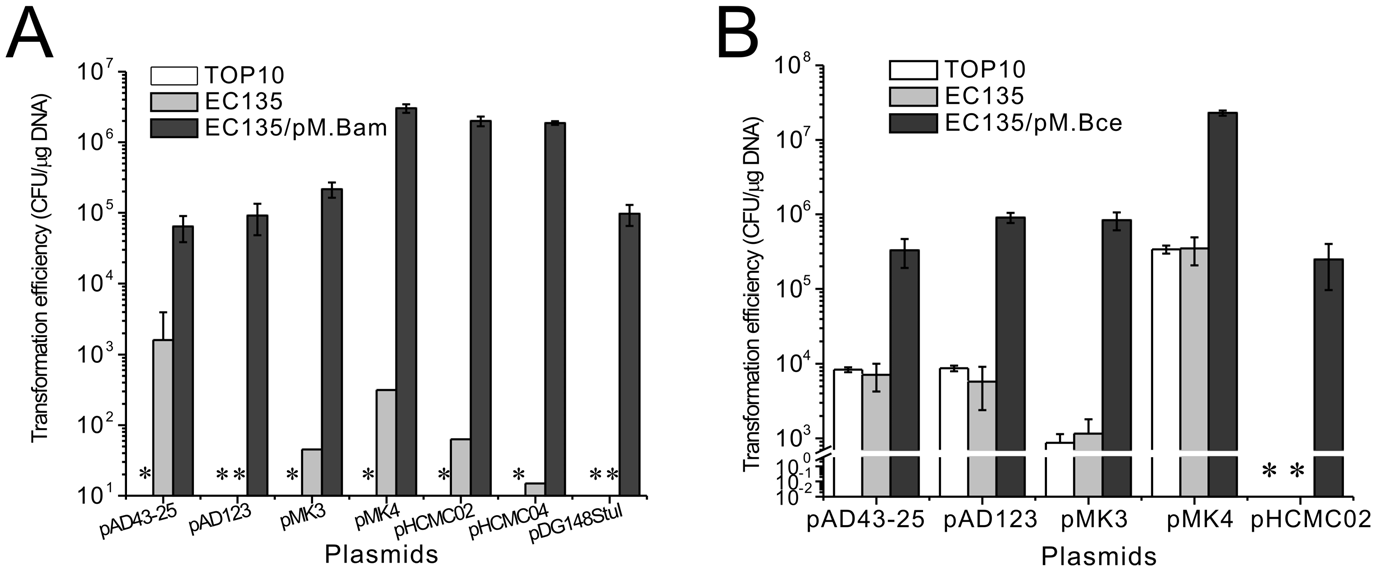
Transformation efficiency of *B. amyloliquefaciens* TA208 and *B. cereus* ATCC 10987 with various shuttle plasmids. (A) Transformation efficiency of the *B. amyloliquefaciens* TA208 strain with various shuttle plasmids prepared from the *E. coli* TOP10, EC135 and the EC135 harboring pM.Bam. (B) Transformation efficiency of the *B. cereus* ATCC 10987 strain with various shuttle plasmids prepared from the *E. coli* TOP10, EC135 and the EC135 harboring pM.Bce. Transformation efficiencies shown are averages of at least three replicates ± SD. * Not Detected.

The *B. amyloliquefaciens* TA208 strain could not be transformed with plasmids prepared from *E. coli* TOP10 cells but could be transformed with plasmids from the *E. coli* EC135 strain with low efficiency; this result indicates that a methylation-dependent Type IV R-M system may exist in *B. amyloliquefaciens* TA208, although its coding gene was not found during annotation of the genome sequence [16]. Hence it may also be that the plasmids methylated at the Dam and Dcm sites would not be inherited in *B. amyloliquefaciens* TA208, *e.g.*, methylated replication origin would not be bound by the replication protein. The MoDMP protocol increased the transformation efficiencies of all the plasmids tested in *B. amyloliquefaciens* TA208. The pMK4 plasmid from MoDMP hosts showed the highest transformation efficiency (3×10^6^ CFU/µg DNA), representing a 10^4^-fold increase compared to that of the plasmids from the *E. coli* EC135 strain. The MoDMP procedure also enabled two previously untransformable plasmids, pAD123 and pDG148StuI, to be transformed at an efficiency of 1×10^5^ CFU/µg DNA (Figure 5A).

For the *B. cereus* ATCC 10987 strain, the MoDMP pipeline increased the transformation efficiency of the pMK4 plasmid to 2×10^7^ CFU/µg DNA and increased the transformation efficiency of pMK3 by 10^3^ fold compared to those from strains *E. coli* TOP10 or EC135 (Figure 5B). The plasmids prepared from *E. coli* TOP10 and EC135 strains showed similar transformation efficiencies, indicating that the putative Type IV R-M systems (BCE_1016 and BCE_2317) in *B. cereus* ATCC 10987 may be inactive. These results were the same as those obtained in the *B. cereus* ATCC 14579 strain, which could be transformed by methylated DNA (DNA from non-*dam dcm* mutant strains) [24], though some researchers prefer to use unmethylated DNA [25].

The high transformation efficiency achieved with the MoDMP method in both *Bacillus* strains would allow for the direct inactivation of genes using non-replicative integration plasmids.

### Gene inactivation using MoDMP integration plasmids in *B. amyloliquefaciens* TA208

**Figure 6:**
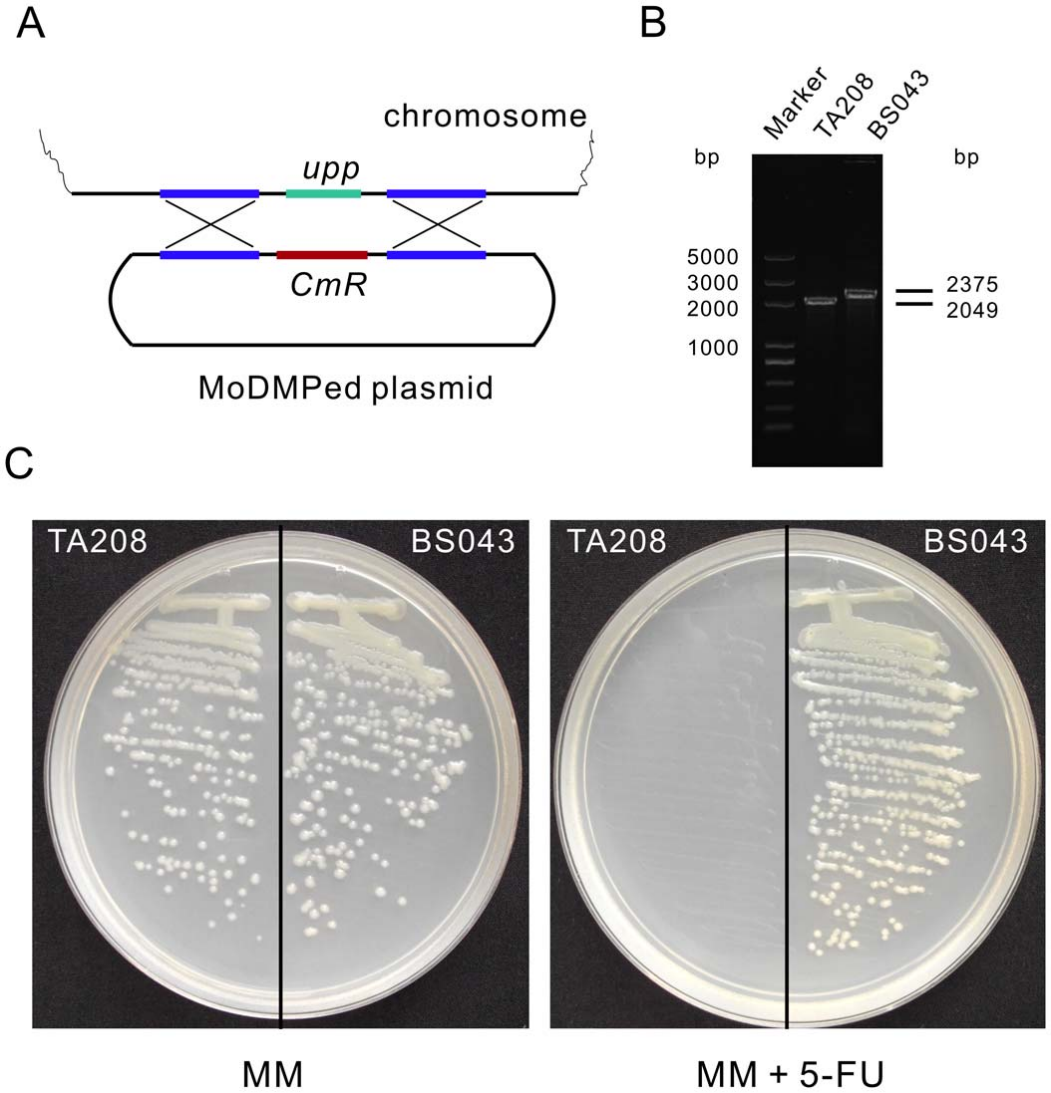
Inactivation of *upp* in *B. amyloliquefaciens* TA208 using an integration plasmid that underwent the MoDMP pipeline. (A) Schematic representation of the recombination event. The MoDMP prepared pWYE748 plasmid will recombine with the chromosome without being degraded by the REases. (B) PCR analysis of the *B. amyloliquefaciens* TA208 strain and the *upp* mutant BS043 strain using primers WB605 and WB606. (C) Verification of the loss of uracil phosphoribosyltransferase activity in *B. amyloliquefaciens* BS043. Strain BS043 could grow on MM plates containing 10 µM 5-FU while the TA208 strain could not. MM without 5-FU was used as a control medium.

To further validate the efficacy of the MoDMP procedure, the gene coding for uracil phosphoribosyltransferase (*upp*) in *B. amyloliquefaciens* TA208 was selected for inactivation using non-replicative integration plasmids. The *B. amyloliquefaciens* TA208 strain was transformed with pWYE748 plasmids that had been through the MoDMP host. The pWYE748 plasmid recombines with chromosome of *B. amyloliquefaciens* TA208 at the *upp* locus with a low rate (10^−6^) because it lacks a replication origin for *Bacillus* (Figure 6A). BS043 was obtained and PCR and sequencing analyses revealed the successful replacement of the *upp* gene with the chloramphenicol resistance gene in this strain (Figure 6B). Uracil phosphoribosyltransferase converts 5-fluorouracil (5-FU) to 5-fluoro-UMP, which is ultimately metabolized to the toxic compound 5-fluoro-dUMP capable of inhibiting the activity of thymidylate synthetase. The *upp*/5-FU module has been widely used in many bacterial species for deletion of genes without introducing antibiotic resistance markers [26]. In contrast with the *B. amyloliquefaciens* TA208 strain, the BS043 strain could grow on minimal medium (MM) supplemented with 5-FU (Figure 6C). These findings suggest that the MoDMP system elevated transformation efficiencies of exogenous plasmid to enable direct gene inactivation, and the *upp* gene could be used as a counter-selection marker for the in-frame deletion of genes in *B. amyloliquefaciens*.

### Genetic transformation of *Nitrobacter* using the MoDMP pipeline

**Figure 7:**
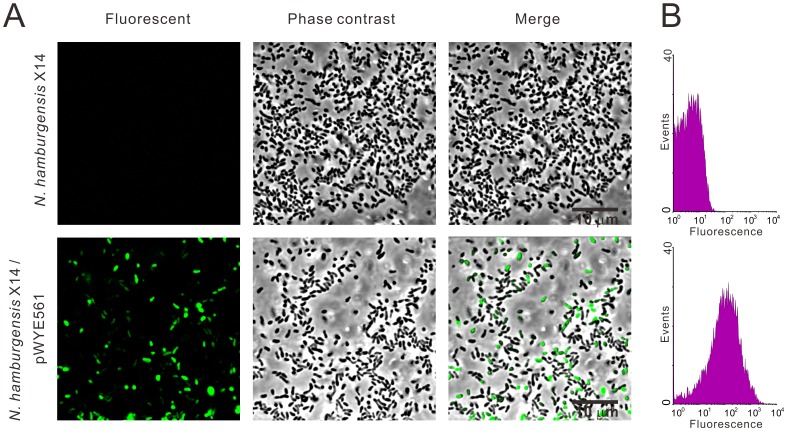
Expression of GFP in *N. hamburgensis* X14. The *N. hamburgensis* X14 strain was transformed with pWYE561 prepared from a MoDMP host. (A) Green fluorescence was observed in transformed *N. hamburgensis* X14 (lower row), but not in untransformed bacteria (upper row). (B) Determination of the proportion of transformed bacteria by flow cytometry analysis (lower). Bacterial culture of the *N. hamburgensis* X14 strain was used as a negative control.

*N. hamburgensis* X14 harbors 11 putative R-M systems, and successful genetic transformation of this strain has not been reported [27]. In this study, the *N. hamburgensis* X14 strain was transformed with plasmids carrying the Green Fluorescent Protein (GFP) encoding gene *gfpmut3a* using the MoDMP procedure. Total genomic DNA was extracted from 10 mL of the transformed bacteria cells. The plasmid was rescued to *E. coli* TOP10 cells, and subsequent plasmid preparation (Figure S5A) and restriction digestion with SalI and PstI (Figure S5B) verified the existence of pWYE561 in the transformed bacterial cell lines. During the subculture process, the bacterial cell lines were monitored for contamination by microscopy and culturing on LB plates at 30°C, and no contamination was observed. Green fluorescent signals were observed in the cytoplasm of *Nitrobacter*, thereby revealing the successful transformation of *Nitrobacter* (Figure 7A).

The culture may contain multiclonal cell lines because the transformants were enriched twice through successive sub-inoculation of the transformation cell mixture in liquid culture (see Materials and Methods for details). Using flow cytometry, the ratio of fluorescent cells was determined to be 50.37% (Figure 7B), demonstrating that 50.37% of the cells were positive transformants. Clonal cell lines could be obtained by streaking the transformant-enriched culture on nylon membranes placed on solid medium and periodically transferred to fresh plates, as described by Sayavedra-Soto *et al.* in *Nitrosomonas europaea* [28].

### Specificity identification for the MTases and the DNA methylation patterns comparison between MoDMP hosts and difficult-to-transform bacteria

To make the MTase expression vectors more useful, the modification sequences of MTases were determined when expressed individually or co-expressed. As shown in Figure S6A, BAMTA208_6525 protected plasmid from cleavage by BamHI (GGATCC), BglII (AGATCT), and partially from BclI (TGATCA), indicating that BAMTA208_6525 modifies RGATCY and partial TGATCA sequences. BAMTA208_6715 protected pMK4 from cleavage by HaeIII (GGCC), Fnu4HI (GCNGC) and Bsp1286I (GDGCHC), and BAMTA208_19835 and BAMTA208_16660 protect pMK4 from TseI (GCWGC) and BamHI (GGATCC) cleavage, respectively. When co-expressed, the four active MTases from *B. amyloliquefaciens* TA208 could protect the plasmids from cleavage by all of the REases tested in the individual expression experiments. However, DNA from the *B. amyloliquefaciens* TA208 strain was only resistant to BamHI cleavage and partially resistant to Fnu4HI and TseI cleavage (Figure S6B), indicating that the expression of BAMTA208_16660 in the native strain was complete, whereas those of BAMTA208_6715 and BAMTA208_19835 were incomplete, and BAMTA208_6525 was not expressed.

For the *B. cereus* ATCC 10987 strain, BCE_0393 could protect plasmid from cleavage by at least 12 REases, *i.e.*, Fnu4HI (partial), TseI, BbvI (GCAGC), HaeIII, EaeI (YGGCCR), HpaII (CCGG), MspI (CCGG, partial), NlaIV (GGNNCC), BssHII (GCGCGC), HhaI (GCGC, partial), AvaII (GGWCC) and PspGI (CCWGG, partial), and the modification sequences of BCE_0393 were concluded as GCWGC, GGCC, CCGG, GGNNCC, GCGCGC, GGWCC and CCWGG (partial). BCE_0365 protected DNA from cleavage by TseI and BbvI, indicating that it modifies GCWGC sequence, BCE_4605 protect DNA from cleavage by AvaII via modification of GGWCC sequence, and BCE_5606 and BCE_5607 both protect DNA from cleavage by BceAI [ACGGC(N)_12/14_] (Figure S7A). These results are consistent with the reports of Xu *et al.* [15], except for that “GGWCC” was added to the modification sequences of BCE_0393 in this study. The multi-specificity nature of the prophage MTase BCE_0393 and its sequence overlapping with other MTases from *B. cereus* ATCC 10987 indicated that it plays a major role in the MoDMP pipeline of this strain.

The pMK4 plasmids prepared from the *E. coli* strain expressing BCE_0392 was challenged with various REases which might be sensitive to m6A modification, including AvaII, BamHI, BbvI, BceAI, BglII, BsiEI, Bsp1286I, BspDI, BstNI, BspHI, DpnII, EaeI, EcoRI, Fnu4HI, HincII, HindIII, HpaII, HinfI, NlaIV, PstI, PshAI, PspGI, SalI, ScrFI, SwaI, SpeI, TaqI and TseI, but resistance to cleavage was not observed. Therefore BCE_0392 might modify sequences that are not recognized by these REase, and new techniques like single-molecule DNA sequencing other than restriction analysis using commercialized REases should be useful in identifying the sequence specificity of BCE_0392 [29]. DNA nicking-associated concatenation activity was also detected for BCE_0392 *in vivo* [15], suggesting that this ParB-Methyltransferase might participate in phage DNA replication or phage packaging, since BCE_0392 was located in a prophage region in the chromosome of the *B. cereus* ATCC 10987 strain [11].

When co-expressed, BCE_0393, BCE_0365, BCE_4605, BCE_5606 and BCE_5607 protected all of the pMK4 plasmid from AvaII and BceAI digestion, protected most of the pMK4 plasmids from Fnu4HI, TseI, BbvI, HaeIII, EaeI, HpaII and NlaIV cleavage, provided pMK4 partial protection from HhaI digestion and provided pHCMC05 full protection from BssHII cleavage (Figure S7B). However, pMK4 plasmid prepared from the *B. cereus* ATCC 10987 strain was only resistant to TseI, BbvI, AvaII and BceAI digestion, and partially resistant to Fnu4HI digestion (Figure S7B), indicating that BCE_0393 is not completely expressed in its native host.

For *N. hamburgensis* X14, Nham_0569 could protect DNA from cleavage by at least 10 REases sensitive to m6A modification, *i.e.*, DpnI (GAmTC), DpnII (GATC), PvuII (CAGCTG), SspI (AATATT), SpeI (ACTAGT), MfeI (CAATTG), NlaIII (CATG), AseI (ATTAAT), HinfI (GANTC) and TfiI (GAWTC), but full protection was not achieved (Figure S8A). Therefore, Nham_0569 might be a multi-specific enzyme harboring at least eight modification sites, *i.e.*, GATC, CAGCTG, AATATT, ACTAGT, CAATTG, CATG, ATTAAT and GANTC, or a new member of the recently characterized non-specific DNA adenine MTase [30]. Nham_3225 protected DNA from HinfI and TfiI cleavage by modifying GANTC sequence (Figure S8A). The pMK4 plasmids prepared from the *E. coli* EC135 strains expressing Nham_0582 and Nham_0803 were not resistant to the cleavage by DpnII, EcoRI, DraI, PvuII, SspI, HinfI, HindIII, BspHI, BamHI, SpeI, KpnI, SacI or ApaLI, and the specificity of Nham_0582 and Nham_0803 was not identified.

The four active MTases from *N. hamburgensis* X14 provided DNA partial protection from cleavage by DpnI, DpnII, PvuII, SspI, SpeI, MfeI, NlaIII, AseI, HinfI and TfiI when co-expressed, and the genomic DNA of the *N. hamburgensis* X14 strain was sensitive to DpnII digestion, partially resistant to DpnI digestion and resistant to SpeI, AseI, HinfI and TfiI digestion (Figure S8B). These results indicated that Nham_0569 was only partially expressed in its native host.

The modification sequences of MTases are summarized to Table 1. The MoDMP hosts and difficult-to-transform bacteria showed similar DNA methylation patterns based on the REase digestion analysis, but the DNA from MoDMP hosts have more modification sites than corresponding difficult-to-transform bacteria. And this was mainly caused by the limited expression of some MTases in their native hosts, especially some prophage-derived MTases, *i.e.*, BAMTA208_6525, BCE_0393 and Nham_0569, which are multi-specific MTases.

## Discussion

Genetic transformation of bacteria harboring multiple R-M systems has been problematic using conventional methods. It has been long recognized that the exogenous MTases over-expressed in *E. coli* could modify DNA *in vivo* and protect them from digestion by their cognate REases [31]. Strategies based on this fact have been developed to overcome the restriction barrier of bacteria, including *in vitro* or *in vivo* plasmid modification prior to transformation [32], [33], heat inactivation of the REases [34] or gene knock-outs [35]. However, it has been reported that the inactivation of the SauI Type I R-M system is insufficient for *Staphylococcus aureus* to efficiently accept foreign DNA [36]. In this study, a strategy has been developed to mimic the DNA methylation patterns of the difficult-to-transform bacteria in a modified *E. coli* strain. To achieve this goal, active MTases from the difficult-to-transform bacteria were co-expressed in an *E. coli* host lacking all of the characterized R-M systems and orphan MTases.

The protocol for genetic transformation of difficult-to-transform bacteria using a plasmid prepared in a different *E. coli* host is diagramed in Figure S9. As indicated in strain *B. amyloliquefaciens* TA208, DNA from the *E. coli* hosts with Dam and Dcm contains methylated bases in GAmTC and CCmWGG sequences but is not methylated at the recognition sequences of the host Type I–III REases; this DNA would then be recognized by Type I–IV REases in the target bacteria (upper left panel in Figure S9). Plasmids prepared from *dam dcm EcoKI* mutant *E. coli* would make the strains transformable at a low efficiency due to the plasmids being able to avoid restriction by the Type IV REases in the target bacterium (lower left panel in Figure S9). Many bacterial species have been reported to restrict DNA containing Dam and Dcm methylation; for example, *B. anthracis* could be transformed by DNA from an *E. coli dam dcm* mutant strain but not by DNA from *E. coli* host strains with the wild type alleles [37], [38]. Additionally, DNA prepared from the *E. coli* SCS110 strain was more accessible to *Corynebacterium glutamicum* than DNA from *E. coli* hosts with Dam and Dcm [39]. However, not all difficult-to-transform bacteria behave like this. Bacteria lacking functional Type IV REases could be transformed by DNA prepared from *E. coli* hosts with Dam, Dcm or EcoKI, albeit at a low efficiency. Currently, it has been shown that the *B. cereus* ATCC 10987 strain does not restrict DNA with Dam and Dcm methylation. It has also been reported that the plasmids methylated in *E. coli* TOP10 cells using the MTases of the target bacteria can allow for the genetic manipulation of *Bifidobacterium breve* [40]. Ryan *et al.* showed that the *bbe02* and *bbq67* loci limited the transformation of *Borrelia burgdorferi* by shuttle vector DNA prepared from *E. coli*, irrespective of its Dam, Dcm or EcoKI methylation status [41]. The *N. hamburgensis* X14 strain used in this study may restrict DNA with Dcm methylation; the plasmid-borne putative Type IV R-M system Nham_4502-Nham_4503 has been annotated in the REBASE database [5], though its activity and specificity remain unclear.

As shown in the upper right panel in Figure S9, expression of exogenous MTases in *E. coli* would result in methylation of chromosomal DNA, and Mrr, McrA and McrBC would recognize and cleave the DNA with foreign patterns, making the strain inviable or resulting in poor MTase expression [42], [43]. Therefore, an *E. coli* strain lacking all of the known R-M systems and orphan MTases was generated with MTases expressed. The plasmids prepared from this host could escape the REases that recognize unmethylated DNA or DNA methylated in foreign patterns (lower right panel in Figure S9). The MoDMP concept could greatly improve genetic transformation efficiency.

Recently, four REases from the *B. cereus* ATCC 10987 strain have been cloned and characterized, namely BceSI, BceSII, BceSIII and BceSIV [15]. Only faint and non-specific hybridization blots were observed using the antibodies against m6A and m5C for the MTase of BceSI (BCE_1018) in this study; these faint blots may be caused by the vagaries of dot blot approaches. It might also be that BCE_1018 modifies the DNA in a way other than methylation, such as hydroxymethylation or glucosyl-hydroxymethylation. BCE_1018 was not included in the downstream MoDMP application. Nevertheless, the highest transformation efficiency was achieved using the plasmids modified by six other MTases and was within the acceptable range for gene knock-out experiments (10^7^ CFU/µg DNA). This efficiency may be caused by the low abundance and the weak REase activity of BceSI in strain *B. cereus* ATCC 10987, as described by Hegna *et al.* [23]. BceSI was induced only when the strain *B. cereus* ATCC 10987 was grown in the presence of exogenous DNA.

Nham_3845 (NhaXI) has been reported to be a fused enzyme harboring both restriction and modification subunits, but the m6A or m4C modification activity was not detected [44]; in this study the MTase activity was not detected either. It might be that Nham_3845 modified DNA in ways other than methylation, which could not be detected using immunoblot assays.

The contribution of individual MTases to genetic transformation was not evaluated in this study because a shuttle plasmid containing all of the MTase recognition sequences cannot be defined. A MTase that does not modify one particular plasmid might be useful when other plasmids are to be used. Therefore, to make a universal system for all plasmids, all of the identified active MTases were employed for MoDMP. The orphan MTase BAMTA208_06715, which lacks a counterpart REase, was also used in the MoDMP pipeline of *B. amyloliquefaciens* TA208. Orphan MTases, such as CcrM, may participate in methylation-directed DNA mismatch repair [45]. Methylated DNA could potentially escape inspection from the host mismatch repair machinery and eventually exhibit an elevated transformation performance, hence the use of BAMTA208_06715 in the MoDMP pipeline.

The use of DNA mimic protein Ocr (overcome classical restriction) alongside the plasmid (TypeOne Restriction Inhibitor, Epicentre; [46]) which specifically inhibits Type I REase activity, also enhances transformation efficiency in bacterial species [47]. Combination of this method with the MoDMP pipeline could further elevate transformation performance in strains which are difficult to transform. Recently, a novel R-M system has been shown to phosphorothioate DNA, preventing the degradation of the DNA by its REase counterparts [48]. The MoDMP concept may also be adapted to those bacteria restricting unphosphorothioated DNA.

In conclusion, we devised a system in *E. coli* that mimics the DNA methylation patterns of bacterial strains harboring multiple R-M systems. Eventually, the R-M barrier of three represented bacterial strains were overcome, including Gram positive, Gram negative, chemoheterotrophs and chemoautotrophs. The adaptability of this pipeline to different bacterial groups suggests a universal potential. This protocol is very fast; a MoDMP plasmid can be generated in less than one week using the *S. cerevisiae* assembler, if the MTase activity assay step is omitted and the putative MTases are cloned and expressed directly. We expect that the pipeline will be applicable to other strains of known genome sequence that are resistant to genetic transformation.

## Materials and Methods

### Strains and plasmids

The strains *E. coli* TOP10 and EC135 were used for the cloning and expression of the MTases. *S. cerevisiae* DAY414 was used for *in vivo* assembly of the MTase genes. The plasmid pBAD43 was used for the cloning and expression of individual MTases, and pWYE724 was used for co-expression of multiple MTases. Several *E. coli*-*Bacillus* shuttle plasmids were used for MoDMP procedure evaluation purposes in the *B. amyloliquefaciens* TA208 and *B. cereus* ATCC 10987 strains. Inactivation of *upp* in *B. amyloliquefaciens* TA208 was performed with pWYE748. Expression of the GFP variant *gfpmut3a* in *N. hamburgensis* was carried out using pBBR1-MCS5. The strains and plasmids used in this study are listed in Table S1.

### Cloning and expression of putative MTases

Putative MTase encoding genes were retrieved from the REBASE database [5]. Genes were PCR amplified and ligated into pBAD43. Individual genes that encode the methylation and specificity subunits of BCE_0839–BCE_0842 system were joined to operons using Splicing by Overlapping Extension (SOE) PCR. All recombinant plasmids were verified by sequencing before use. The *E. coli* EC135 strain was transformed with pBAD43 plasmids encoding MTase genes. Single colonies were used to inoculate LB medium and cultured until an OD_600_ reading of 0.2 was reached, and then arabinose was added to a final concentration of 0.2% to induce MTase expression. Expression was induced overnight at 30°C.

### Southwestern dot blot assay for methylation activity

The DNA methylation activity of the putative MTases was analyzed using a southwestern dot blot assay as described previously [18]. Total genomic DNA from the *E. coli* EC135 strains expressing individual or multiple MTases was prepared using a DNeasy Blood and Tissue Kit (Qiagen). DNA concentrations were determined using a Nanodrop 2000C spectrophotometer (Thermo Scientific). The DNA was then denatured at 100°C for 3 min and immediately cold shocked in an ice-water bath. Samples were spotted onto Protran BA85 nitrocellulose membrane (Whatman) and fixed by UV cross-linking. The membrane was blocked in 5% non-fat milk and incubated with rabbit antisera against DNA containing m6A at a dilution of 1∶10,000 (New England Biolabs), rabbit antisera against m4C at a dilution of 1∶10,000 (New England Biolabs), or a mouse monoclonal antibody against m5C diluted 1∶20,000 (Zymo Research). After washing, the membrane was incubated with secondary goat anti-rabbit or anti-mouse antibodies conjugated with horseradish peroxidase (HRP) (Jackson ImmunoResearch) at a dilution of 1∶10,000. The blots were visualized using the ECL prime Western blotting detection reagent (GE Healthcare), and DNA methylation signals were exposed to Kodak X-Ray film.

For quantification of the hybridization signals, the films were scanned and the gray scale of the spots was quantified using Quantity One (Bio-Rad). After normalization, the values were plotted as bar charts.

### LC–MS assays for m6dA

To obtain the nucleoside samples of genomic DNA for LC-MS analysis, 30 µg of DNA prepared from the *E. coli* EC135 strain or strains expressing MTases were digested to deoxynucleosides with 50 U of DNA Degradase Plus (Zymo Research); the digestion was carried out in 100 µL volume at 37°C for 18 h. The m6dA standard was purchased from Santa Cruz Biotechnology.

The characterization of m6dA was performed on an Agilent 6520 Accurate-Mass QTOF LC/MS system (Agilent Technologies) equipped with an electrospray ionization (ESI) source. 30 µL of the samples were injected to the Agilent 1200 HPLC using an Agilent Zorbax Extend-C_18_ 1.8 µm 2.1×50 mm column with the column temperature kept at 35°C. Water with 0.1% formic acid and methanol were used as mobile phases A and B, respectively, with a flow rate of 0.2 mL/min. The following gradient was used: 0% B for 3.0 min, increase to 60% B in 4.5 min, 60–95% B over 2.5 min, 95% B for 5 min, and then decreased to 0% B over 0.5 min prior to re-stabilization of 14.5 min before the next injection.

The MS data were collected in positive ionization mode with nitrogen supplied as the nebulizing and drying gas. The temperature of the drying gas was set at 300°C. The flow rate of the drying gas and the pressure of the nebulizer were 600 L/h and 25 psi, respectively. The fragmentor and capillary voltages were kept at 90 and 3,500 V, respectively. Full-scan spectra were acquired over a scan range of *m/z* 80–1000 at 1.03 spectra/s.

### One-step assembly of multiple active MTase genes

Multiple MTase genes were rapidly assembled by taking advantage of the high DNA recombination activity in *S. cerevisiae* [49]. The CEN6 replicon was added to pBAD43 followed by TRP1 allele from pDDB78 at the ClaI site to yield pWYE724; the addition of these elements enables replication and screening in *S. cerevisiae*. The active MTase genes were amplified using PCR primers that contained 50 bp of overlapping sequence to the adjacent gene from their corresponding pBAD43 plasmids. *S. cerevisiae* DAY414 was transformed with the DNA fragments encoding the active MTases from the individual bacterial strains and the pWYE724 plasmid linearized at the EcoRI and SalI loci. *S. cerevisiae* DAY414 was then selected for tryptophan autotrophy on synthetic complete (SC) medium lacking tryptophan. *S. cerevisiae* transformation was performed using the lithium acetate method [50]. Plasmids were rescued into *E. coli* TOP10 cells as described by Robzyk *et al* [51]. All recombinant plasmids were verified by restriction digestion and DNA sequencing before subsequent use. The plasmids carrying multiple MTase genes from *B. amyloliquefaciens* TA208, *B. cereus* ATCC 10987 and *N. hamburgensis* X14 were named pM.Bam, pM.Bce and pM.Nham, respectively.

### Construction of integration plasmids and GFP–expressing plasmids

The homologous DNA sequences flanking the *upp* gene of *B. amyloliquefaciens* TA208 (641 bp upstream and 669 bp downstream) and the chloramphenicol resistance gene of pMK4 were amplified and joined using SOE-PCR. This cassette was ligated into the pMD19-T vector (Takara) and verified by DNA sequencing. The resulting plasmid was named pWYE748.

A 216 bp promoter region of the Nham_3450 gene was PCR amplified from the genome of the *N. hamburgensis* X14 strain and joined to *gfpmut3a* by SOE-PCR. The resulting GFP expression cassette was ligated into pBBR1-MCS5 at the SalI and PstI sites to yield pWYE561.

### *In vivo* methylation of plasmids

Various shuttle and integrative plasmids were transformed into the *E. coli* EC135 strains carrying MTase encoding genes. MTase expression was then induced by incubation with 0.2% arabinose at 30°C to allow the *in vivo* methylation of these plasmids.

### Transformation of difficult-to-transform bacteria

Transformation of *B. cereus* ATCC 10987 was carried out as described previously with the following modifications [24]. The *B. cereus* ATCC 10987 strain was cultured in LB medium until the culture reached an OD_600_ of 0.2 and was then incubated on ice for 10 min. Cells were harvested by centrifugation at 8,000 g at 4°C for 10 min. After washing four times with ice-cold transformation buffer (10% sucrose, 15% glycerol, 1 mM Tris-HCl, pH 8.0), the electro-competent cells were resuspended in 1/125 volume of the original culture. The cells (90 µL) were mixed with 100 ng of the column-purified plasmids and loaded into a pre-chilled 1 mm gap cuvette. After a brief incubation on ice, the cells were shocked with a 2.1 kV pulse generated by a BTX ECM399 electroporator (Harvard Apparatus). The cells were immediately diluted with 1 mL NCMLB medium (17.4 g/L K_2_HPO_4_, 11.6 g/L NaCl, 5 g/L glucose, 10 g/L tryptone (Oxoid), 5 g/L yeast extract (Oxoid), 0.3 g/L trisodium citrate, 0.05 g/L MgSO_4_·7H_2_O, 69.2 g/L mannitol and 91.1 g/L sorbitol, pH 7.2) and incubated at 37°C for 3 h to allow the expression of the antibiotic resistance genes. Aliquots of the recovery mix were spread onto LB plates supplemented with 5 µg/mL chloramphenicol or 10 µg/mL kanamycin and cultured overnight at 37°C.

Electroporation of *B. amyloliquefaciens* TA208 was performed using the combined cell-wall weakening and cell-membrane fluidity disturbing procedure described previously [17].

The *N. hamburgensis* X14 strain was grown in DSMZ 756a medium (1.5 g/L yeast extract, 1.5 g/L peptone (BD Biosciences), 2 g/L NaNO_2_, 0.55 g/L sodium pyruvate, 1 mL/L trace element solution (33.8 mg/L MnSO_4_⋅H_2_O, 49.4 mg/L H_3_BO_3_, 43.1 mg/L ZnSO_4_⋅7H_2_O, 37.1 mg/L (NH_4_)_6_Mo_7_O_24_, 97.3 mg/L FeSO_4_⋅7H_2_O and 25 mg/L CuSO_4_⋅5H_2_O) and 100 mL/L stock solution (0.07 g/L CaCO_3_, 5 g/L NaCl, 0.5 g/L MgSO_4_⋅7H_2_O, 1.5 g/L KH_2_PO_4_), pH 7.4) at 28°C in the dark until reaching an OD_600_ of 0.1. The cells were then harvested by centrifugation at 8000 g at 4°C for 10 min and washed four times with ice-cold 10% glycerol. The cells were resuspended in 10% glycerol at a 1,000-fold greater concentration compared to that of the original culture volume. The cell suspension (90 µL) was mixed with 150 ng of the pWYE561 plasmid and electroporated with an ECM399 electroporator at 1.2 kV. The cells were washed into 100 mL 756a medium and recovered at 28°C with gentle shaking for one day. The bacteria were then grown in the presence of 20 µg/mL gentamycin for one day. The bacterial culture was used at a ratio of 1∶100 to inoculate fresh 756a medium containing antibiotics and was shaken at 180 rpm at 28°C. After about three weeks, the culture became turbid. The bacterial culture was subcultured once more to enrich for transformed cells and took one week to reach an OD_600_ of 0.1. The culture was tested for contamination microscopically and by streaking the culture onto LB plates. Successful transformation of strain X14 was verified by plasmid preparation using the Plasmid Mini Kit (OMEGA Bio-tek), PCR amplification of *gfpmut3a* and plasmid rescue. Expression of GFP was observed using a Leica TCS SP2 confocal laser scanning microscope (Leica Microsystems), and the ratio of fluorescent cells was determined using a BD FACS Calibur flow cytometer (BD Biosciences).

### Inactivation of *upp* in *B. amyloliquefaciens* TA208

The pWYE748 plasmid was transformed into the *E. coli* EC135 strain harboring pM.Bam. After induction of MTase expression, 1 µg of the pWYE748 plasmid was transferred to *B. amyloliquefaciens* TA208, and the cells were selected for chloramphenicol resistance. Positive clones were verified by PCR and sequencing using primers (WB605 and WB606) specific to the flanking sequences of the homologous arms. The *upp* knock-out strain *B. amyloliquefaciens* BS043 was validated by growth on MM plates supplemented with 10 µM 5-FU [26] and 100 mg/L adenosine.

All of the PCR primers used in this study are listed in Table S2.

### Determination of the modification sequences of MTases

The modified plasmid DNA was challenged by the cognate REases to determine the modification sequences of the cloned MTases. To facilitate the identification, the high-copy plasmid pMK4 was transformed to *E. coli* EC135 harboring individual or multiple MTase genes, and *in vivo* methylated pMK4 plasmids were prepared and challenged by the cognate REases after linearization by REases that have sole cutting sites in pMK4 (NcoI, EcoRI, SpeI or BamHI). The pMK4 plasmids prepared from *E. coli* EC135, and the plasmids from *B. amyloliquefaciens* TA208 or *B. cereus* ATCC 10987 was used as the negative and positive controls in the experiments of individual MTase and multiple MTases, respectively. The plasmids pWYE690 and pHCMC02 were tested for their resistance to BclI cleavage conferred by BAMTA208_6525 when it was expressed individually and co-expressed due to the lack of BclI site in pMK4. For the same reason, pWYE699 and pHCMC05 were used in testing the protection conferred by BCE_0393 from BssHII cleavage.

Since the broad-host-range plasmid derivative pWYE561 showed a low copy number in *E. coli*, pMK4 was also used in identification of the modification sites of the MTases from strain *N. hamburgensis* X14. Genomic DNA of the strain was used as a control for co-expressed MTases.

## Supporting Information

Figure S1 Generation and verification of the *E. coli* strain EC135. (A) PCR analysis of *dcm* deletion in the *E. coli* EC067 strain using primers WB064 and WB065; *E. coli* TOP10 strain was used as a control. (B) Digestion of chromosomal DNA with BstNI and PspGI for verification of *dcm* inactivation in the *E. coli* EC067, EC132 and EC135 strains; *E. coli* TOP10 strain was used as a control. (C) Survival ratio of *E. coli* EC067 and EC132 (*recA*+) strains when challenged with nalidixic acid (NA). (D) PCR analysis of *dam* deletion in the *E. coli* EC135 strain using primers WB062 and WB063; The *E. coli* EC132 strain was used as a control. (E) DpnI and DpnII digestion of chromosomal DNA for verification of *dam* inactivation in the *E. coli* EC135 strain; *E. coli* TOP10, EC067 and EC132 strains were used as controls. (F) Sensitivity of the *E. coli* EC135 strain to 2-aminopurine (2-AP) exposure; *E. coli* TOP10, EC067 and EC132 strains were used as controls.
(TIF)

Figure S2 Plots of hybridization intensity in dot blots. The spots of dot blot assay for individual MTases (Figure 1) were quantified and plotted. (A) Hybridization intensity of the DNA *in vivo* methylated by the MTases from the *B. amyloliquefaciens* TA208 and *B. cereus* ATCC 10987 strains. For m6A, signals of the spots were normalized to that of 150 ng of *E. coli* TOP10 DNA (100%); for m4C, signals of the spots were normalized to that of 150 ng of *E. coli* EC135 DNA expressing BAMTA208_06525 (100%); for m5C, signals of the spots were normalized to that of 150 ng of *E. coli* EC135 DNA expressing BAMTA208_06715 (100%). (B) Hybridization intensity of the DNA *in vivo* methylated by the MTases from *N. hamburgensis* X14. For m6A, signals of the spots were normalized to that of 150 ng of *E. coli* TOP10 DNA (100%); for m4C, signals of the spots were normalized to that of 150 ng of M.BamHI *in vivo* methylated *E. coli* EC135 DNA (100%); for m5C, signals of the spots were normalized to that of 150 ng of M.AluI *in vivo* methylated *E. coli* EC135 DNA (100%). Relative intensities shown are averages of three replicates ± SD.
(TIF)

Figure S3 Growth curve of *E. coli* EC135 strains expressing Nham_0569, Nham_0803 and Nham_3225. The *E. coli* EC135 strains harboring pWYE712 (Nham_0569), pWYE714 (Nham_0803) and pWYE719 (Nham_3225) were cultured until an OD_600_ reading of 0.2 was reached, and then arabinose was added to a final concentration of 0.2% to induce MTase expression. The *E. coli* EC135 strain harboring the empty vector pBAD43 was used as the control.
(TIF)

Figure S4 Assembly of MTase genes. (A) MoDMP plasmids visualized on agarose gels. (B) EcoRV digestion of pM.Bam. (C) HindIII digestion of pM.Bce. (D) BamHI digestion of pM.Nham. The digestion patterns of the pM.Bam, pM.Bce and pM.Nham plasmids correspond with the theoretical patterns (B–D).
(TIF)

Figure S5 Rescue of pWYE561 from transformed *N. hamburgensis* X14. (A) Original pWYE561 plasmid from *E. coli* TOP10 and rescued pWYE561 from *N. hamburgensis* X14. (B) Original and rescued pWYE561 digested by SalI and PstI. The length of pBBR1MCS5 backbone and promoter-*gfp*mut3a is noted.
(TIF)

Figure S6 The modification sequences analysis of MTases from strain *B. amyloliquefaciens* TA208 expressed individually and co-expressed. (A) Analysis of the modification sequences of MTases when expressed individually. The pMK4 plasmids prepared from *E. coli* EC135 strains expressing individual MTases (identified in the text above the gel images) was challenged by cognate REases (identified in the text above the gel images). In each two-lane-grouped REase case, unmethylated pMK4 plasmids from *E. coli* EC135 were used as the control in the left lane, and methylated pMK4 plasmids prepared from the EC135 strains expressing individual MTases were used in the right lane. Prior to REase challenging, the pMK4 plasmids were linearized by REases (identified beneath the gel images) with sole recognition sites. In the test of BclI site modified by BAMTA208_06525, EcoRI-linearized pWYE690 plasmid was challenged by BclI, and mock-treated linearized pWYE690 was used as the control. (B) Confirmation of the modification sequences of MTases when co-expressed. pMK4 plasmids prepared from the *E. coli* EC135 strain harboring pM.Bam (left lane in each REase case) or from the *B. amyloliquefaciens* TA208 strain (right lane in each REase case) were challenged by cognate REases. In the BclI case, pHCMC02 from *E. coli* EC135 and the strain harboring pM.Bam were challenged, with the genomic DNA of the *B. amyloliquefaciens* TA208 strain used as the control. M, DNA marker; U, undigested. The proportion of plasmids resistant to REase digestion is marked by asterisks; the proportion of plasmids partially digested by REases is marked by arrowheads.
(TIF)

Figure S7 The modification sequences analysis of MTases from strain *B. cereus* ATCC 10987 expressed individually and co-expressed. (A) Analysis of the modification sequences of MTases expressed individually. The pMK4 plasmids prepared from the *E. coli* EC135 strains expressing individual MTases (identified in the text above the gel images) was challenged by cognate REases (identified in the text above the gel images). In each two-lane-grouped REase case, unmethylated pMK4 plasmids from *E. coli* EC135 were used as the control in the left lane, and methylated pMK4 plasmids prepared from the EC135 strains expressing individual MTases were used in the right lane. Prior to REase challenging, the pMK4 plasmids were linearized by REases (identified beneath the gel images) with sole recognition sites in it. In the test of BssHII site modified by BCE_0393, SpeI-linearized pWYE699 plasmid was challenged by BssHII, and mock-treated linearized pWYE699 was used as the control. (B) Confirmation of the modification sequences of MTases when co-expressed. The pMK4 plasmids prepared from the *E. coli* EC135 strain harboring pM.Bce (left lane in each REase case) or from *B. cereus* ATCC 10987 (right lane in each REase case) were challenged by cognate REases. In the BclI case, pHCMC05 from *E. coli* EC135 and the strain harboring pM.Bce were challenged, and the genomic DNA of *B. cereus* ATCC 10987 was used as the control. M, DNA marker; U, undigested. The proportion of plasmids resistant to REase digestion is marked by asterisks; the proportion of plasmids partially digested by REases is marked by arrowheads.
(TIF)

Figure S8 The modification sequences analysis of MTases from *N. hamburgensis* X14 expressed individually and co-expressed. (A) Analysis of the modification sequences of MTases expressed individually. The pMK4 plasmids prepared from the *E. coli* EC135 strains expressing individual MTases (identified in the text above the gel images) was challenged by cognate REases (identified in the text above the gel images). In each two-lane-grouped REase case, unmethylated pMK4 plasmids from *E. coli* EC135 were used as the control in the left lane, and methylated pMK4 plasmids prepared from the EC135 strains expressing individual MTases were used in the right lane. Prior to REase challenging, the pMK4 plasmids were linearized by REases (identified beneath the gel images) with sole recognition sites in it. (B) Confirmation of the modification sequences of MTases when co-expressed. The pMK4 plasmids prepared from *E. coli* EC135 (left lane in each REase case) or the strain EC135 harboring pM.Nham (right lane in each REase case) were challenged by cognate REases. The genomic DNA of *N. hamburgensis* X14 was used as the controls. M, DNA marker; U, undigested. The proportion of plasmids resistant to REase digestion is marked by asterisks.
(TIF)

Figure S9 Model of how the MoDMP protocol results in the transformation of difficult-to-transform bacteria. Plasmid DNA prepared from an *E. coli* host with Dam, Dcm and EcoKI are methylated at GAmTC, CCmWGG and EcoKI recognition sequences, but not at the recognition sequence of the REases from the difficult-to-transform bacteria. This DNA would be degraded by Type I–IV REases upon transformation into the bacteria (upper-left quarter). Expression of exogenous MTases from the difficult-to-transform bacteria strains in the *E. coli* host with McrA, McrBC and Mrr would cause degradation of the host's chromosomal DNA (upper-right quarter). Plasmids prepared from *dam*- *dcm*- *EcoKI*- *E. coli* hosts can be transformed at low efficiencies because the restriction imposed by Type IV REases has been relieved (lower-left quarter). When the plasmids were prepared from the *E. coli* strain lacking known R-M systems and orphan MTases while expressing MTases of the bacteria, the plasmids showed similar modification patterns to the bacteria and can be transformed at high efficiencies (lower-right quarter).
(TIF)

Table S1 Strains and plasmids used in this study.
(DOC)

Table S2 Primers used for PCR.
(DOC)

Text S1 Supporting Information Methods and Supporting Information Results. The construction and validation of *E. coli* EC135 is described in detail.
(DOC)
