# Supplementary figures and images for: A Mimicking-of-DNA-Methylation-Patterns Pipeline for Overcoming the Restriction Barrier of Bacteria
Source: PLoS Genet. 2012 Sep 27;8(9):e1002987. doi: 10.1371/journal.pgen.1002987 (PMC3459991; doi:10.1371/journal.pgen.1002987)

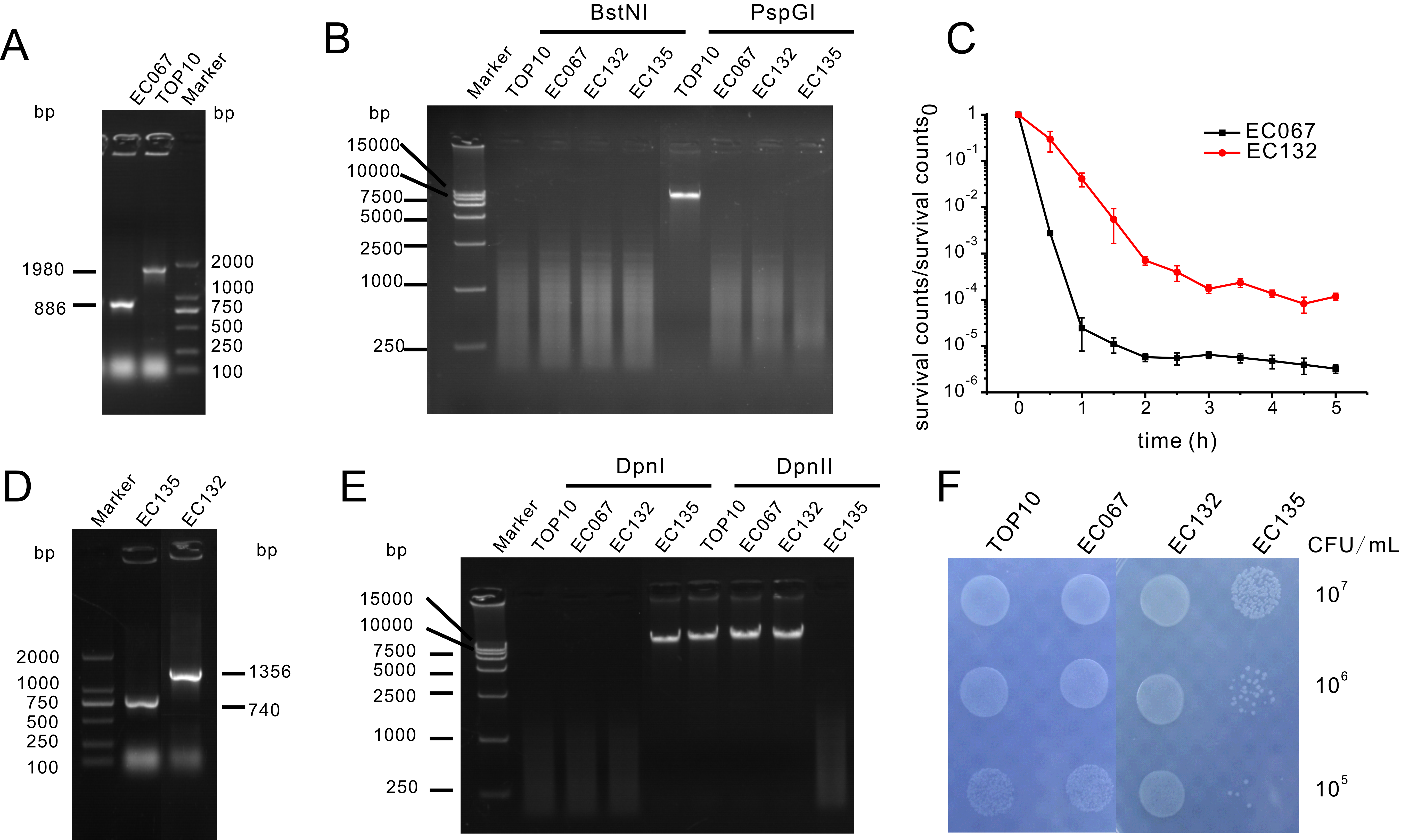

Supplement: Figure S1 — Generation and verification of the E. coli strain EC135. (A) PCR analysis of dcm deletion in the E. coli EC067 strain using primers WB064 and WB065; E. coli TOP10 strain was used as a control. (B) Digestion of chromosomal DNA with BstNI and PspGI for verification of dcm inactivation in the E. coli EC067, EC132 and EC135 strains; E. coli TOP10 strain was used as a control. (C) Survival ratio of E. coli EC067 and EC132 (recA+) strains when challenged with nalidixic acid (NA). (D) PCR analysis of dam deletion in the E. coli EC135 strain using primers WB062 and WB063; The E. coli EC132 strain was used as a control. (E) DpnI and DpnII digestion of chromosomal DNA for verification of dam inactivation in the E. coli EC135 strain; E. coli TOP10, EC067 and EC132 strains were used as controls. (F) Sensitivity of the E. coli EC135 strain to 2-aminopurine (2-AP) exposure; E. coli TOP10, EC067 and EC132 strains were used as controls. (TIF) [file pgen.1002987.s001.tif]

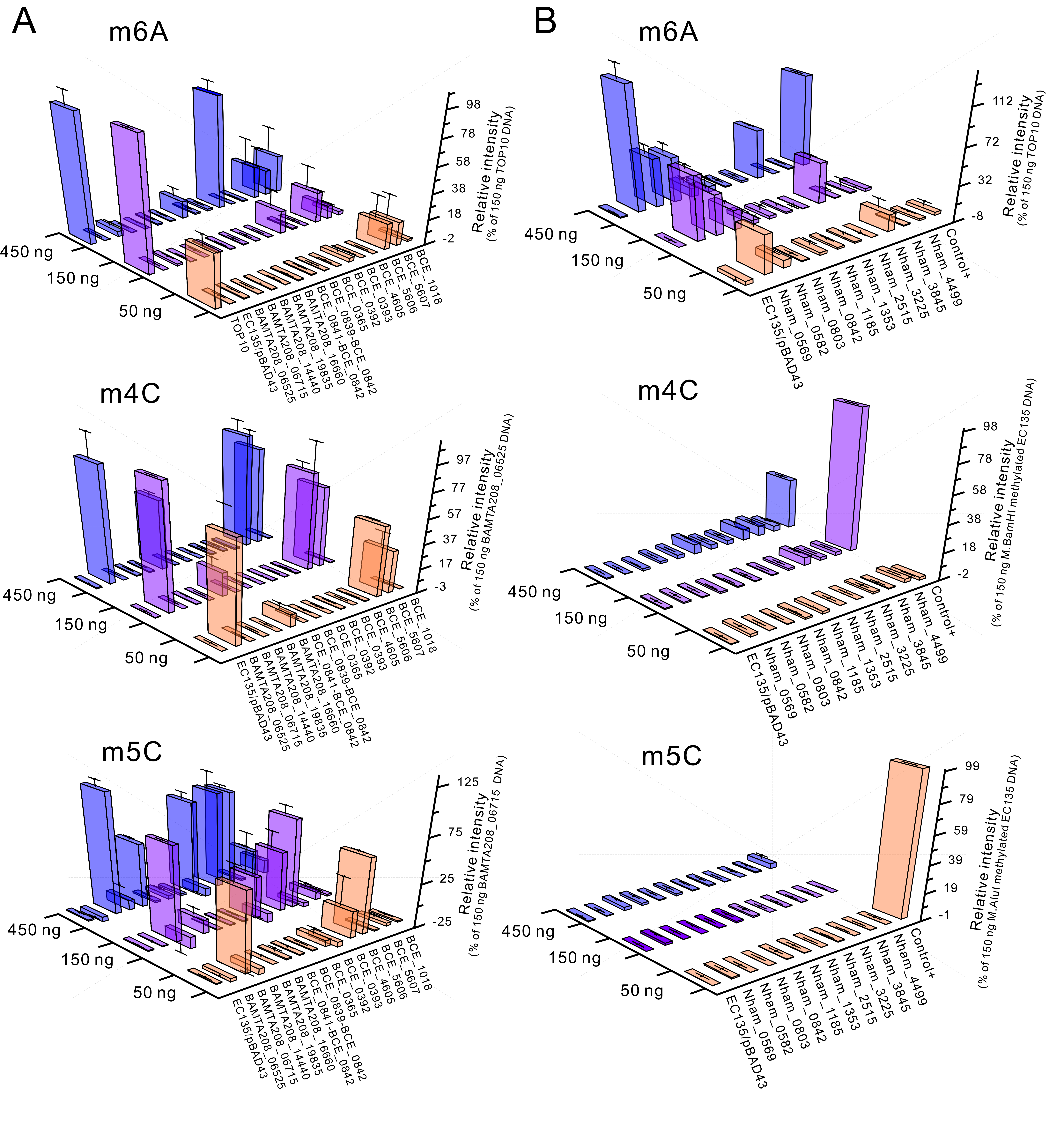

Supplement: Figure S2 — Plots of hybridization intensity in dot blots. The spots of dot blot assay for individual MTases (Figure 1) were quantified and plotted. (A) Hybridization intensity of the DNA in vivo methylated by the MTases from the B. amyloliquefaciens TA208 and B. cereus ATCC 10987 strains. For m6A, signals of the spots were normalized to that of 150 ng of E. coli TOP10 DNA (100%); for m4C, signals of the spots were normalized to that of 150 ng of E. coli EC135 DNA expressing BAMTA208_06525 (100%); for m5C, signals of the spots were normalized to that of 150 ng of E. coli EC135 DNA expressing BAMTA208_06715 (100%). (B) Hybridization intensity of the DNA in vivo methylated by the MTases from N. hamburgensis X14. For m6A, signals of the spots were normalized to that of 150 ng of E. coli TOP10 DNA (100%); for m4C, signals of the spots were normalized to that of 150 ng of M.BamHI in vivo methylated E. coli EC135 DNA (100%); for m5C, signals of the spots were normalized to that of 150 ng of M.AluI in vivo methylated E. coli EC135 DNA (100%). Relative intensities shown are averages of three replicates ± SD. (TIF) [file pgen.1002987.s002.tif]

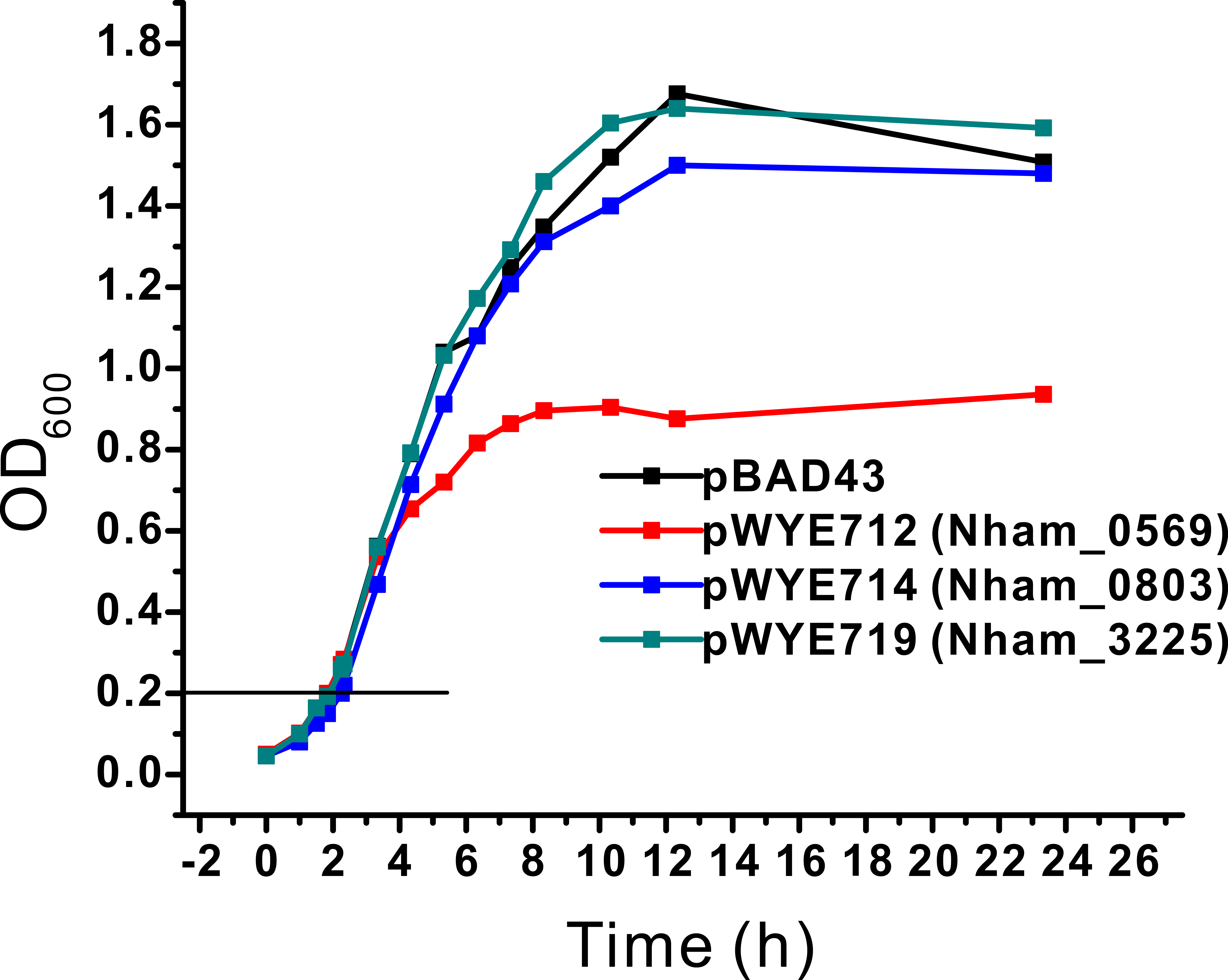

Supplement: Figure S3 — Growth curve of E. coli EC135 strains expressing Nham_0569, Nham_0803 and Nham_3225. The E. coli EC135 strains harboring pWYE712 (Nham_0569), pWYE714 (Nham_0803) and pWYE719 (Nham_3225) were cultured until an OD600 reading of 0.2 was reached, and then arabinose was added to a final concentration of 0.2% to induce MTase expression. The E. coli EC135 strain harboring the empty vector pBAD43 was used as the control. (TIF) [file pgen.1002987.s003.tif]

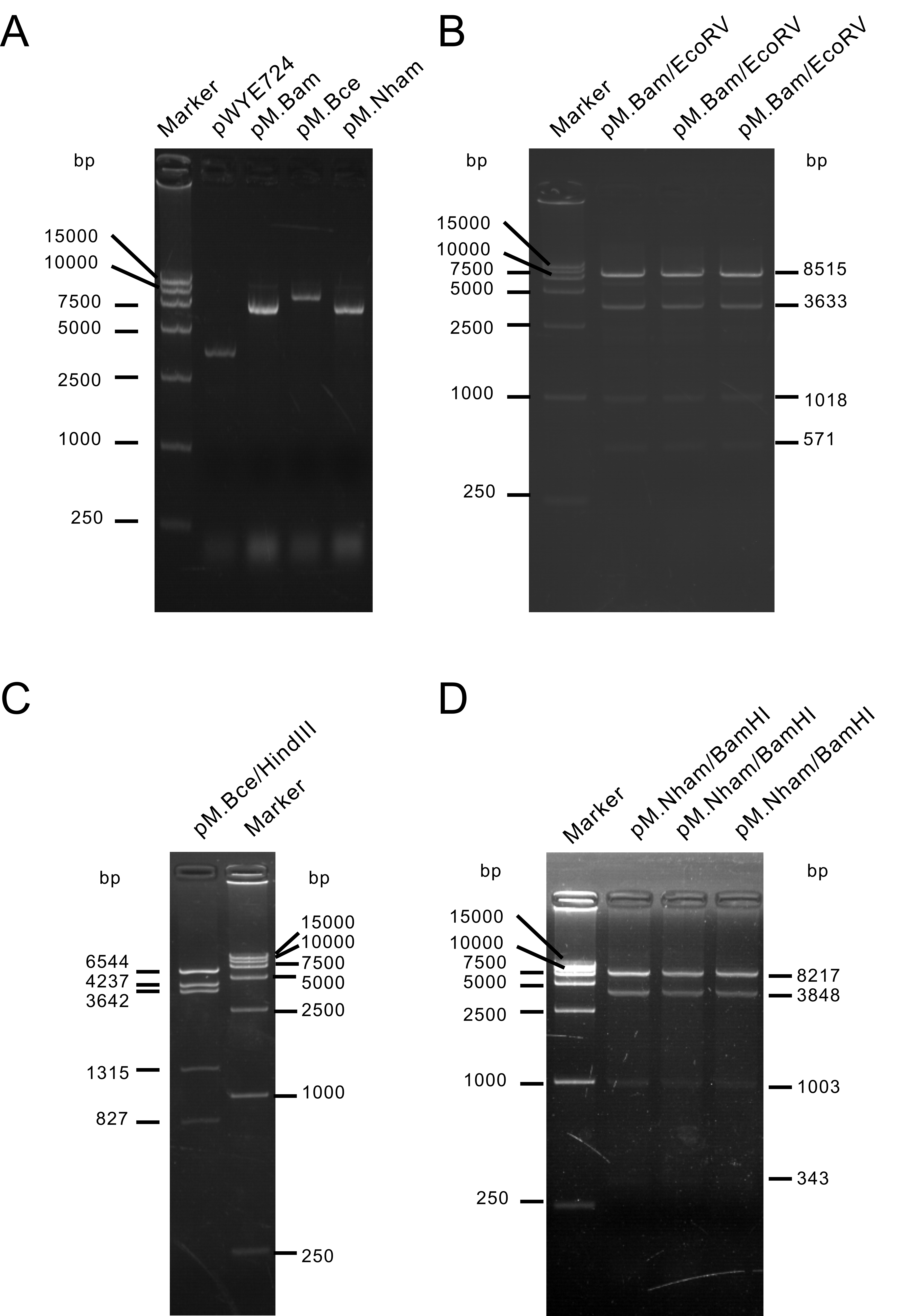

Supplement: Figure S4 — Assembly of MTase genes. (A) MoDMP plasmids visualized on agarose gels. (B) EcoRV digestion of pM.Bam. (C) HindIII digestion of pM.Bce. (D) BamHI digestion of pM.Nham. The digestion patterns of the pM.Bam, pM.Bce and pM.Nham plasmids correspond with the theoretical patterns (B–D). (TIF) [file pgen.1002987.s004.tif]

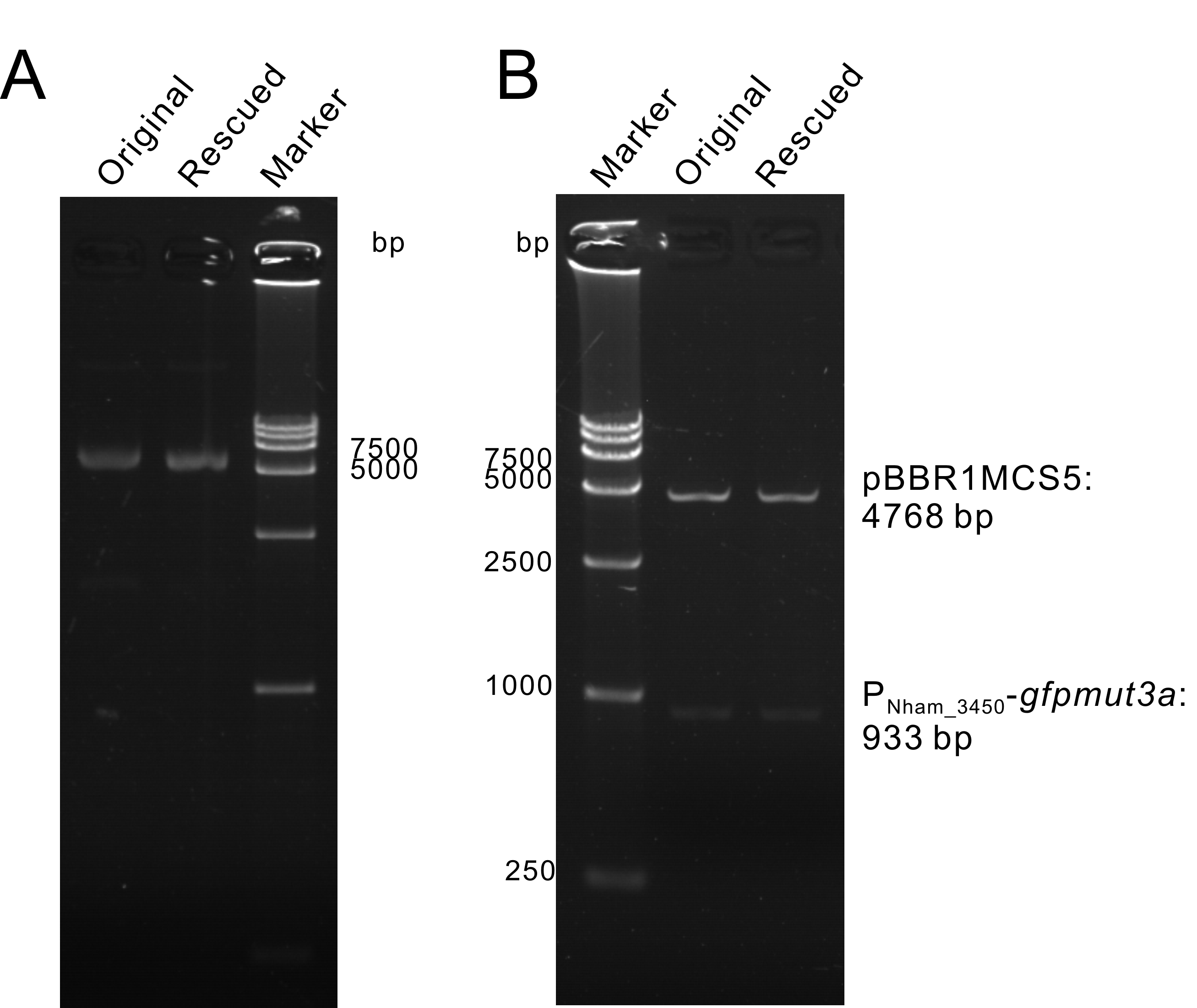

Supplement: Figure S5 — Rescue of pWYE561 from transformed N. hamburgensis X14. (A) Original pWYE561 plasmid from E. coli TOP10 and rescued pWYE561 from N. hamburgensis X14. (B) Original and rescued pWYE561 digested by SalI and PstI. The length of pBBR1MCS5 backbone and promoter-gfpmut3a is noted. (TIF) [file pgen.1002987.s005.tif]

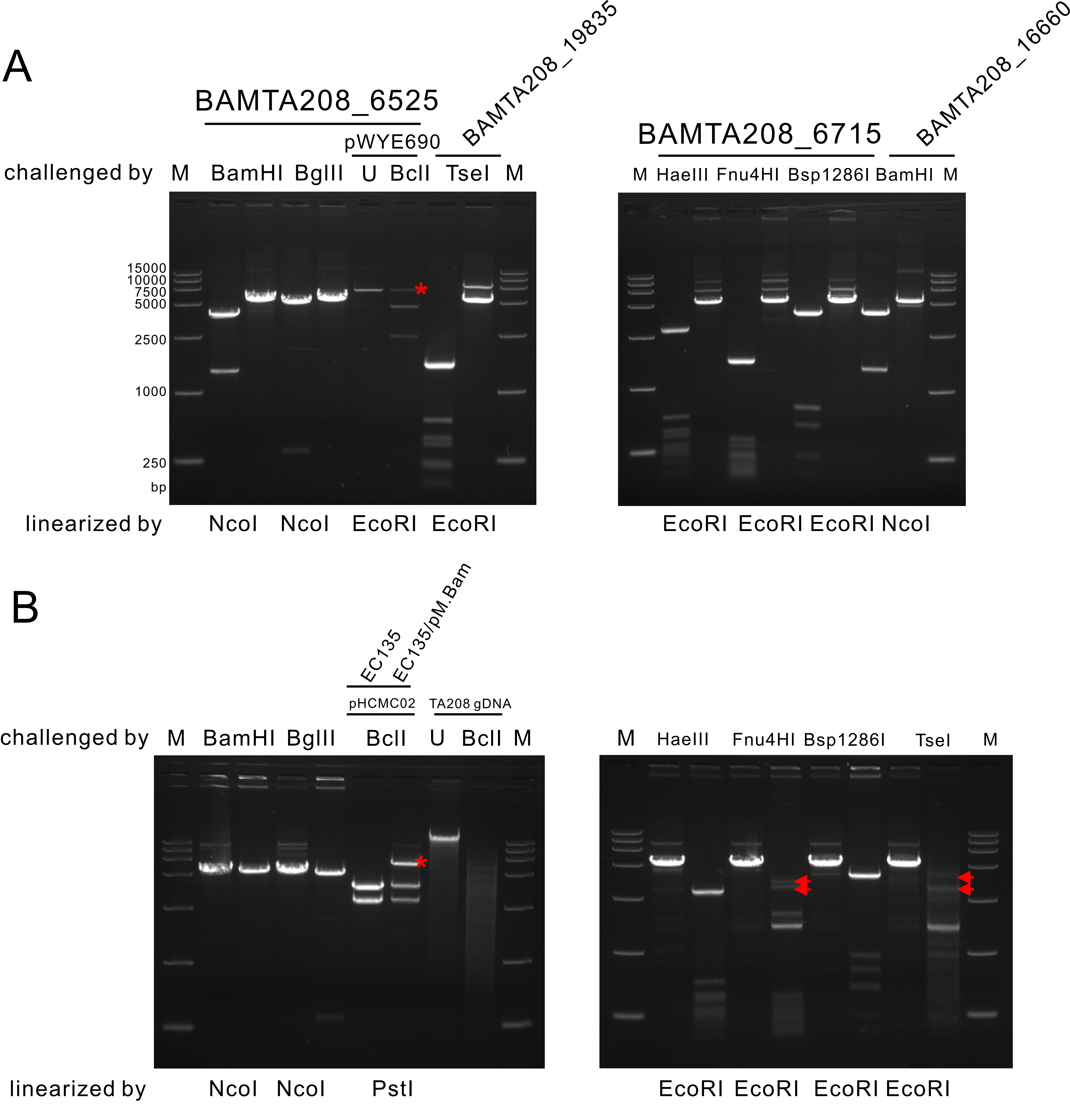

Supplement: Figure S6 — The modification sequences analysis of MTases from strain B. amyloliquefaciens TA208 expressed individually and co-expressed. (A) Analysis of the modification sequences of MTases when expressed individually. The pMK4 plasmids prepared from E. coli EC135 strains expressing individual MTases (identified in the text above the gel images) was challenged by cognate REases (identified in the text above the gel images). In each two-lane-grouped REase case, unmethylated pMK4 plasmids from E. coli EC135 were used as the control in the left lane, and methylated pMK4 plasmids prepared from the EC135 strains expressing individual MTases were used in the right lane. Prior to REase challenging, the pMK4 plasmids were linearized by REases (identified beneath the gel images) with sole recognition sites. In the test of BclI site modified by BAMTA208_06525, EcoRI-linearized pWYE690 plasmid was challenged by BclI, and mock-treated linearized pWYE690 was used as the control. (B) Confirmation of the modification sequences of MTases when co-expressed. pMK4 plasmids prepared from the E. coli EC135 strain harboring pM.Bam (left lane in each REase case) or from the B. amyloliquefaciens TA208 strain (right lane in each REase case) were challenged by cognate REases. In the BclI case, pHCMC02 from E. coli EC135 and the strain harboring pM.Bam were challenged, with the genomic DNA of the B. amyloliquefaciens TA208 strain used as the control. M, DNA marker; U, undigested. The proportion of plasmids resistant to REase digestion is marked by asterisks; the proportion of plasmids partially digested by REases is marked by arrowheads. (TIF) [file pgen.1002987.s006.tif]

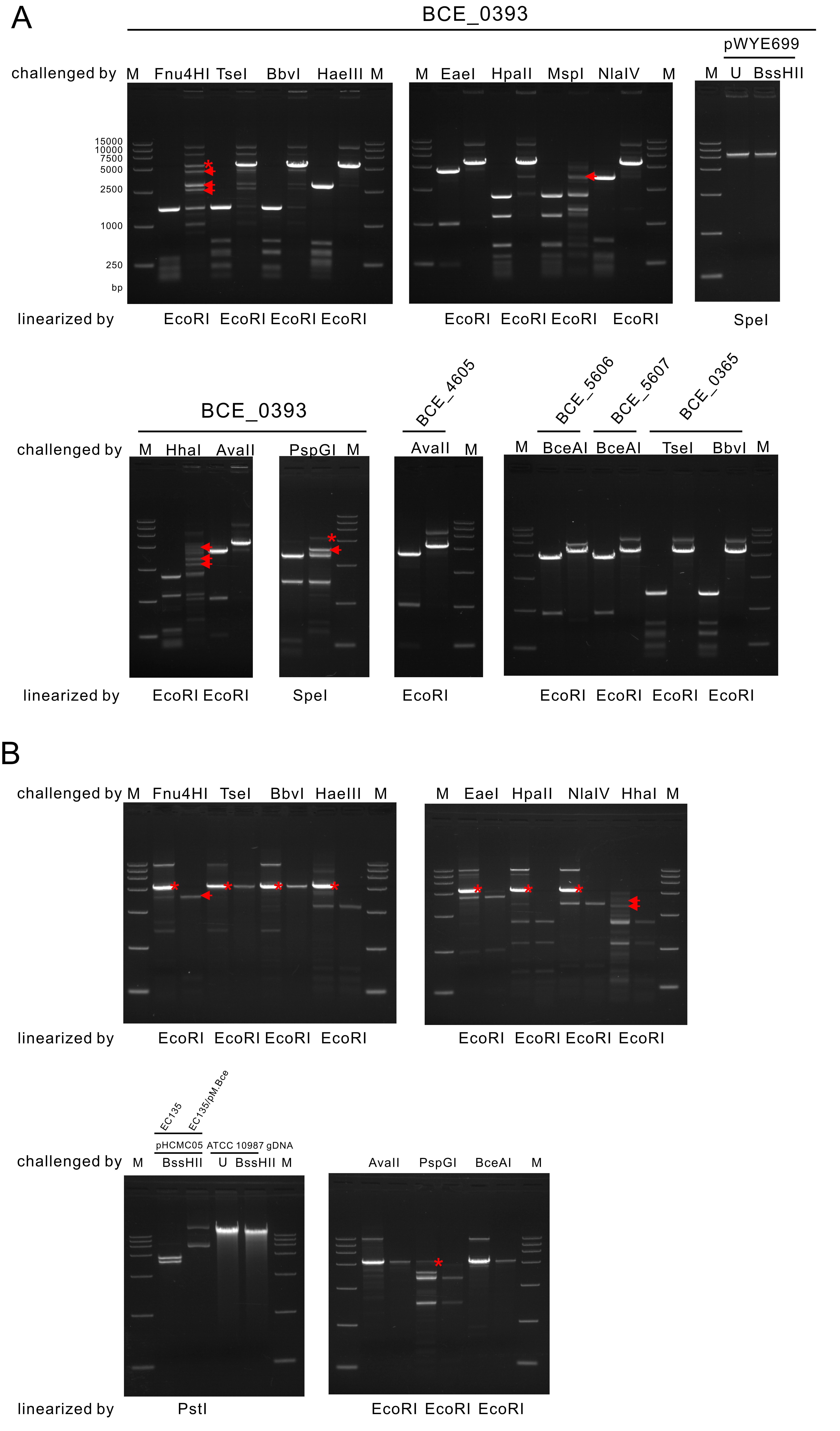

Supplement: Figure S7 — The modification sequences analysis of MTases from strain B. cereus ATCC 10987 expressed individually and co-expressed. (A) Analysis of the modification sequences of MTases expressed individually. The pMK4 plasmids prepared from the E. coli EC135 strains expressing individual MTases (identified in the text above the gel images) was challenged by cognate REases (identified in the text above the gel images). In each two-lane-grouped REase case, unmethylated pMK4 plasmids from E. coli EC135 were used as the control in the left lane, and methylated pMK4 plasmids prepared from the EC135 strains expressing individual MTases were used in the right lane. Prior to REase challenging, the pMK4 plasmids were linearized by REases (identified beneath the gel images) with sole recognition sites in it. In the test of BssHII site modified by BCE_0393, SpeI-linearized pWYE699 plasmid was challenged by BssHII, and mock-treated linearized pWYE699 was used as the control. (B) Confirmation of the modification sequences of MTases when co-expressed. The pMK4 plasmids prepared from the E. coli EC135 strain harboring pM.Bce (left lane in each REase case) or from B. cereus ATCC 10987 (right lane in each REase case) were challenged by cognate REases. In the BclI case, pHCMC05 from E. coli EC135 and the strain harboring pM.Bce were challenged, and the genomic DNA of B. cereus ATCC 10987 was used as the control. M, DNA marker; U, undigested. The proportion of plasmids resistant to REase digestion is marked by asterisks; the proportion of plasmids partially digested by REases is marked by arrowheads. (TIF) [file pgen.1002987.s007.tif]

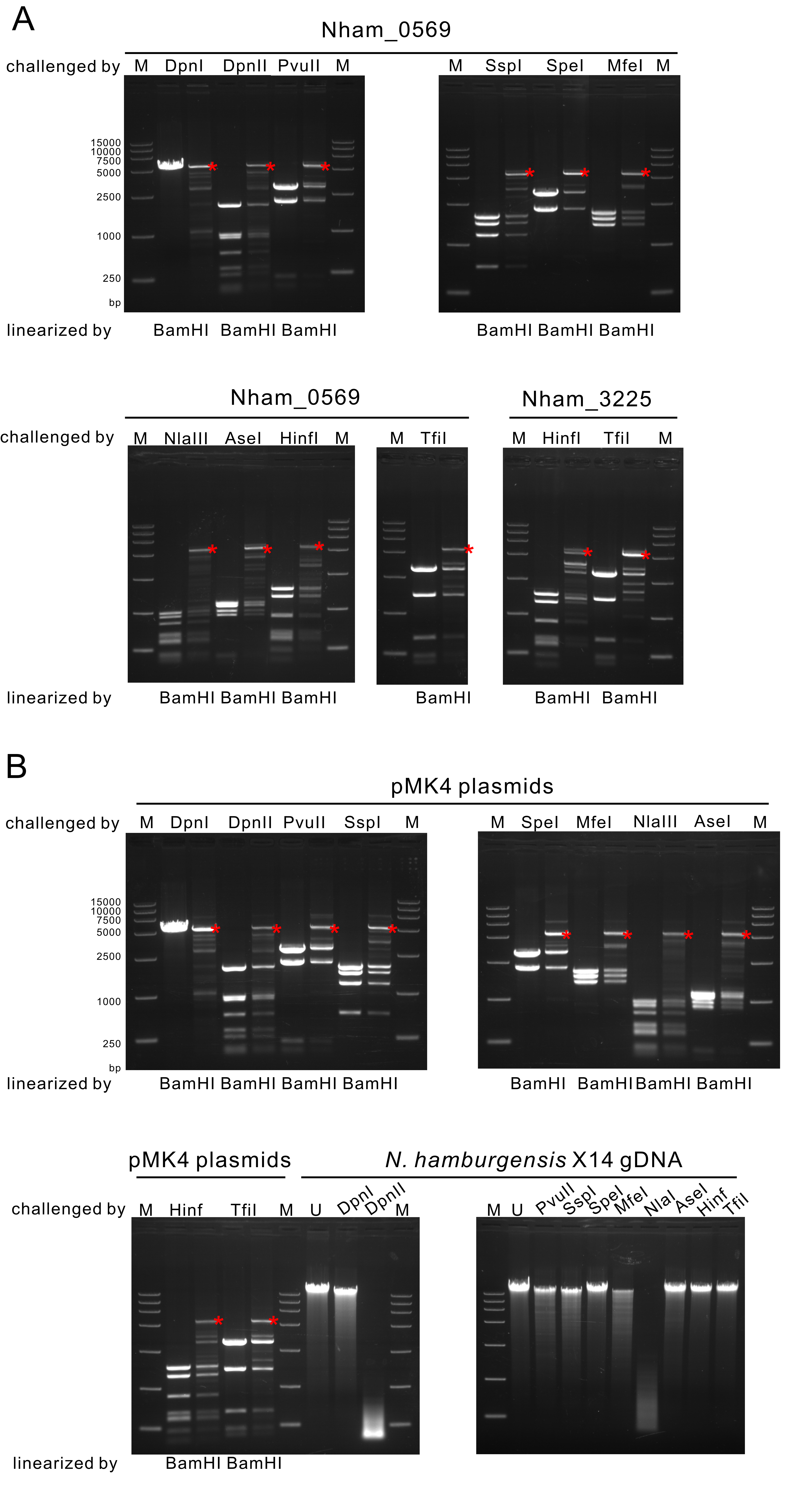

Supplement: Figure S8 — The modification sequences analysis of MTases from N. hamburgensis X14 expressed individually and co-expressed. (A) Analysis of the modification sequences of MTases expressed individually. The pMK4 plasmids prepared from the E. coli EC135 strains expressing individual MTases (identified in the text above the gel images) was challenged by cognate REases (identified in the text above the gel images). In each two-lane-grouped REase case, unmethylated pMK4 plasmids from E. coli EC135 were used as the control in the left lane, and methylated pMK4 plasmids prepared from the EC135 strains expressing individual MTases were used in the right lane. Prior to REase challenging, the pMK4 plasmids were linearized by REases (identified beneath the gel images) with sole recognition sites in it. (B) Confirmation of the modification sequences of MTases when co-expressed. The pMK4 plasmids prepared from E. coli EC135 (left lane in each REase case) or the strain EC135 harboring pM.Nham (right lane in each REase case) were challenged by cognate REases. The genomic DNA of N. hamburgensis X14 was used as the controls. M, DNA marker; U, undigested. The proportion of plasmids resistant to REase digestion is marked by asterisks. (TIF) [file pgen.1002987.s008.tif]

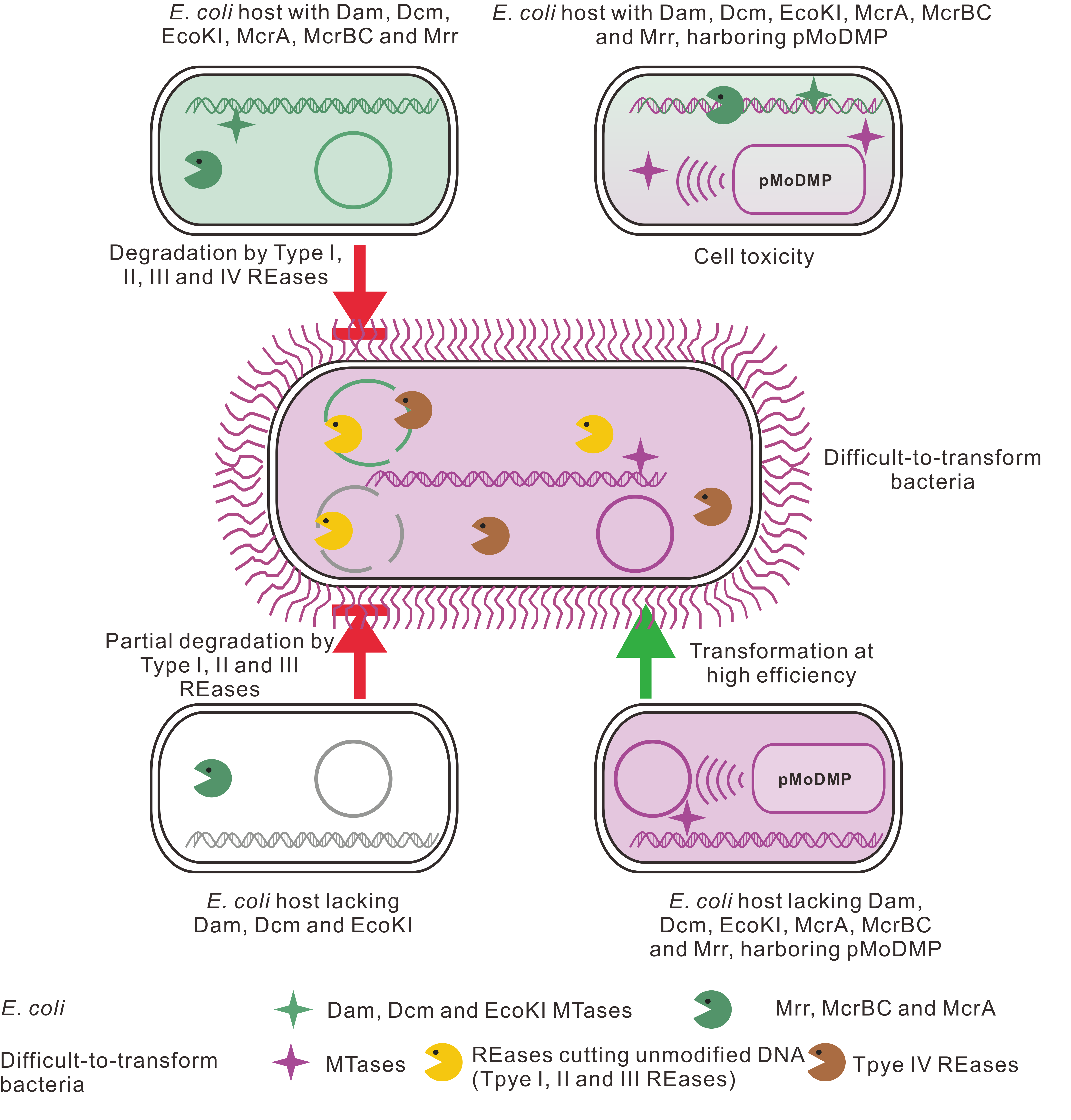

Supplement: Figure S9 — Model of how the MoDMP protocol results in the transformation of difficult-to-transform bacteria. Plasmid DNA prepared from an E. coli host with Dam, Dcm and EcoKI are methylated at GAmTC, CCmWGG and EcoKI recognition sequences, but not at the recognition sequence of the REases from the difficult-to-transform bacteria. This DNA would be degraded by Type I–IV REases upon transformation into the bacteria (upper-left quarter). Expression of exogenous MTases from the difficult-to-transform bacteria strains in the E. coli host with McrA, McrBC and Mrr would cause degradation of the host's chromosomal DNA (upper-right quarter). Plasmids prepared from dam- dcm- EcoKI- E. coli hosts can be transformed at low efficiencies because the restriction imposed by Type IV REases has been relieved (lower-left quarter). When the plasmids were prepared from the E. coli strain lacking known R-M systems and orphan MTases while expressing MTases of the bacteria, the plasmids showed similar modification patterns to the bacteria and can be transformed at high efficiencies (lower-right quarter). (TIF) [file pgen.1002987.s009.tif]
