# Supplementary material for: A Mimicking-of-DNA-Methylation-Patterns Pipeline for Overcoming the Restriction Barrier of Bacteria
Source: PLoS Genet. 2012 Sep 27;8(9):e1002987. doi: 10.1371/journal.pgen.1002987 (PMC3459991; doi:10.1371/journal.pgen.1002987)
Supplement: Table S1 — Strains and plasmids used in this study. (DOC) [file pgen.1002987.s010.doc]

**Table S1.** Strains and plasmids used in this study.

| Strains and plasmids | Characteristics | Reference or source |
| --- | --- | --- |
| *E. coli* strains |  |  |
| *E. coli* TOP10 | R-M genotype: *mcrA* Δ(*mrr*-*hsdRMS*-*mcrBC*) *recA1* | Invitrogen |
| *E. coli* EC067 | TOP10 Δ*dcm*::*FRT* | This study |
| *E. coli* EC132 | EC067 *recA+* | This study |
| *E. coli* EC135 | EC132 Δ*dam*::*FRT,* genotype of R-M systems: *mcrA* Δ(*mrr*-*hsdRMS*-*mcrBC*) Δ*dcm*::*FRT* Δ*dam*::*FRT* | This study |
|  |  |  |
| *Saccharomyces* strain |  |  |
| *S. cerevisiae* DAY414 | Tryptophan auxotrophic | [1] |
|  |  |  |
| Strains for AMoDMP |  |  |
| *B. amyloliquefaciens* TA208 | Guanosine-producing strain | [2] |
| *B. cereus* ATCC 10987 | Wild type | BGSC^a^ |
| *N. hamburgensis* X14 | Wild type, equal to ATCC^b^ 25391 | DSMZ^c^ |
| *B. amyloliquefaciens* BS043 | TA208 Δ*upp*::*Cm^R^* | This study |
| Plasmids |  |  |
| pBAD43 | pSC101 ori, spectinomycine resistance, arabinose inducible promoter | [3] |
| pWYE690 | BAMTA208_6525 cloned into pBAD43 | This study |
| pWYE691 | BAMTA208_6715 cloned into pBAD43 | This study |
| pWYE692 | BAMTA208_14440 cloned into pBAD43 | This study |
| pWYE693 | BAMTA208_19835 cloned into pBAD43 | This study |
| pWYE694 | BAMTA208_16660 cloned into pBAD43 | This study |
| pWYE695 | BCE_0841 and BCE_0842 cloned into pBAD43 | This study |
| pWYE696 | BCE_0839 and BCE_0842 cloned into pBAD43 | This study |
| pWYE697 | BCE_0365 cloned into pBAD43 | This study |
| pWYE698 | BCE_0392 cloned into pBAD43 | This study |
| pWYE699 | BCE_0393 cloned into pBAD43 | This study |
| pWYE700 | BCE_4605 cloned into pBAD43 | This study |
| pWYE701 | BCE_5606 cloned into pBAD43 | This study |
| pWYE702 | BCE_5607 cloned into pBAD43 | This study |
| pWYE703 | BCE_1018 cloned into pBAD43 | This study |
| pWYE712 | Nham_0569 cloned into pBAD43 | This study |
| pWYE713 | Nham_0582 cloned into pBAD43 | This study |
| pWYE714 | Nham_0803 cloned into pBAD43 | This study |
| pWYE715 | Nham_0842 cloned into pBAD43 | This study |
| pWYE716 | Nham_1185 cloned into pBAD43 | This study |
| pWYE717 | Nham_1353 cloned into pBAD43 | This study |
| pWYE718 | Nham_2515 cloned into pBAD43 | This study |
| pWYE719 | Nham_3225 cloned into pBAD43 | This study |
| pWYE720 | Nham_3845 cloned into pBAD43 | This study |
| pWYE721 | Nham_4499 cloned into pBAD43 | This study |
| pWYE724 | pBAD43 derivative, *E. coli*-*S*. *cerevisiae* shuttle plasmid, CEN6 ARS4 ori and TRP1 marker in *S*. *cerevisiae* | This study |
| pM.Bam | pWYE724 carrying active MTases from *B. amyloliquefaciens* TA208: BAMTA208_06525, BAMTA208_06715, BAMTA208_19835 and BAMTA208_16660 | This study |
| pM.Bce | pWYE724 carrying active MTases from *B. cereus* ATCC 10987: BCE_0393, BCE_4605, BCE_5606, BCE_5607, BCE_0365 and BCE_0392 | This study |
| pM.Nham | pWYE724 carrying active MTases from *N. hamburgensis* X14: Nham_0569, Nham_0582, Nham_0803 and Nham_3225 | This study |
| pAD123 | *E. coli*-*Bacillus* shuttle plasmid, chloramphenicol resistance in *Bacillus*, for promoter trapping | BGSC |
| pAD43-25 | pAD123 derivative, *gfpmut3a* controlled by *upp* promoter | BGSC |
| pMK3 | *E. coli*-*Bacillus* shuttle plasmid, replicate in *Bacillus* with pUB110 origin | BGSC |
| pMK4 | *E. coli*-*Bacillus* shuttle plasmid, replicate in *Bacillus* with pC194 origin | BGSC |
| pHCMC02 | *E. coli*-*Bacillus* shuttle plasmid,  replication, featuring high structural stability, carrying *lepA* promoter | BGSC |
| pHCMC04 | pHCMC02 derivative, carrying *xylA* promoter | BGSC |
| pHCMC05 | pHCMC02 derivative, carrying P_spac_ promoter | BGSC |
| pDG148StuI | *E. coli*-*Bacillus* shuttle plasmid, featuring ligation-independent cloning and inducible expression | BGSC |
| pWYE748 | Integration plasmid for *upp* inactivation in *B. amyloliquefaciens*, upp upstream homologous arm - chloramphenicol resistance - *upp* downstream homologous arm cassette ligated into the multiple cloning site of the TA cloning vector pMD19-T | This study |
| pBBR1MCS-5 | Broad-host-range cloning vector, gentamycin resistance | [4] |
| pWYE561 | Promoter region of Nham_3450 and *gfpmut3a* cloned to pBBR1MCS-5 | This study |

^a^ *Bacillus* Genetic Stock Center; ^b^ American Type Culture Collection; ^c^ Deutsche Sammlung von Mikroorganisem und Zellkulturen

**References**

1. Zacchi LF, Gomez-Raja J, Davis DA (2010) Mds3 regulates morphogenesis in *Candida albicans* through the TOR pathway. Mol Cell Biol 30: 3695-3710.

2. Zhang G, Deng A, Xu Q, Liang Y, Chen N, et al. (2011) Complete genome sequence of *Bacillus amyloliquefaciens* TA208, a strain for industrial production of guanosine and ribavirin. J Bacteriol 193: 3142-3143.

3. Guzman LM, Belin D, Carson MJ, Beckwith J (1995) Tight regulation, modulation, and high-level expression by vectors containing the arabinose PBAD promoter. J Bacteriol 177: 4121-4130.

4. Kovach ME, Elzer PH, Steven Hill D, Robertson GT, Farris MA, et al. (1995) Four new derivatives of the broad-host-range cloning vector pBBR1MCS, carrying different antibiotic-resistance cassettes. Gene 166: 175-176.
