# Supplementary material for: A Mimicking-of-DNA-Methylation-Patterns Pipeline for Overcoming the Restriction Barrier of Bacteria
Source: PLoS Genet. 2012 Sep 27;8(9):e1002987. doi: 10.1371/journal.pgen.1002987 (PMC3459991; doi:10.1371/journal.pgen.1002987)
Supplement: Table S2 — Primers used for PCR. (DOC) [file pgen.1002987.s011.doc]

**Table** S2. Primers used for PCR.

| Primers | Sequence (5’-3’) | Notes |
| --- | --- | --- |
| Primers for the construction of the EC135 strain |  |  |
| WB089 | CTAAATGGCTGTAATTATGTTAACCTGTCGGCCATCTCAGATGGCCGGTGAAATCTTTGAGCGATTGTGTAGGCTGGAG | Primers for amplification of the *cat* gene from pKD3, underlined are -56 - -1 and 1219 - 1274 bp of *dcm* |
| WB090 | ACCGGAATACGGAATTTCGCTTCTCCCGGCGCTTCAAAACCCATTAAGCGCGCGCATAACGGCTGACATGGGAATTAGC |
| WB064 | TGCTGAAGCTACCGCAAACCATG | Primers flanking the *dcm* gene, for verification of *dcm* knock-out |
| WB065 | GCACTCCCAGACAATCAATACGC |
| WB253 | ATAAGAATGCGGCCGCCACTTGATACTGTATGAGCATACAG | Primers for cloning *recA*_WT_ into pKOV, NotI and BamHI sites are underlined |
| WB254 | CGCGGATCCCGGGATGTTGATTCTGTCATGGCAT |
| WB087 | CTGGATGCTGTCGGAGCTTTCTCCACAGCCGGAGAAGGTGTAATTAGTTAGTCAGCTTGAGCGATTGTGTAGGCTGGAG | Primers for amplification of the *cat* gene from pKD3, underlined are -56 - -1 and 685-740 bp of *dam* |
| WB088 | ACTTTGACGACATGCAATTTTGCGCGCTGATACCACTCACGCGTTAACATCGTATCTAACGGCTGACATGGGAATTAGC |
| WB062 | GGCCGATCTGAAGTAATCAAGGT | Primers flanking the *dam* gene, for verification of *dam* knock-out |
| WB063 | TCCAGATAGCTCAGAGGTGTCGC |
| Primers for cloning of MTases |  |  |
| WB295 | ATGCACCATGGAACTAAACAAAATACATAATAATG | BAMTA208_06525, NcoI and XbaI sites are underlined |
| WB296 | ATGCATCTAGATCAACTATGTACTTGAGGTAATCGA |
| WB297 | ATGCAGAATTCATGAATAAACTACGAGTAATGAGTC | BAMTA208_6715, EcoRI and XbaI sites are underlined |
| WB298 | ATGCATCTAGATTATTCAGATTCTTTATTATCGTATG |
| WB299 | ATGCAGAATTCATGCAACACGACCATGTCGGTCAG | BAMTA208_14440, EcoRI and XbaI sites are underlined |
| WB300 | ATGCATCTAGATTATTTTTCTTTTTGAATCCATTG |
| WB301 | ATGCAGAATTCATGAACAGCAATAATAAGAGATTT | BAMTA208_19835, EcoRI and SalI sites are underlined |
| WB302 | ATGCAGTCGACTTATTCTAAATCTAATAATTCATT |
| WB303 | ATGCAGAATTCATGCGTTTTTTTTCTGTTTTTGACATTG | BAMTA208_16660, EcoRI and SalI sites are underlined |
| WB304 | ATGCAGTCGACTCATTCTAGACTTAATGAAATTTGG |
| WB305 | ATGCAGCTAGCAAGGCAGGGAAAATAAATGAACAACC | BCE_0841 and BCE_0842, NheI and KpnI sites are underlined |
| WB306 | ATGCAGGTACCTTATTTGATCTTTTCTTTTAATACTTC |
| WB307 | ATGCACCATGGAAACTAATTACGAAAGAAG | BCE_0839 and BCE_0842, NcoI and KpnI sites are underlined. WB308 and WB309 have 25 bp sequence complementary to each other. |
| WB308 | CCTCAGCTTCTTTTATTTTGTCCAATTCATTAGTCCT |
| WB309 | TGGACAAAATAAAAGAAGCTGAGGTGGCTAAGTAATGG |
| WB310 | ATGCAGGTACCTTATTTGATCTTTTCTTTTAATACTTC |
| WB311 | ATGCAGCTAGCAGGAGGGAGAGTGATATTATGATATT | BCE_0365, NheI and KpnI sites are underlined |
| WB312 | ATGCAGGTACCTCATTCTTTAATACTTGGCTCTACG |
| WB313 | ATGCAGCTAGCAGGCGGTGGAAAAGTATGTTGATAG | BCE_0392, NheI and KpnI sites are underlined |
| WB314 | ATGCAGGTACCTTACTCATCCTCATTCACCTCTGGC |
| WB315 | ATGCAGCTAGCCAGAGGTGAATGAGGATGAGTAAATTG | BCE_0393, NheI and KpnI sites are underlined |
| WB316 | ATGCAGGTACCTTAATCGGCGGTATTTTGTGTAGATAAC |
| WB317 | ATGCAGCTAGCAGGAGGTAAGACATATGAACTGTATT | BCE_4605, NheI and KpnI sites are underlined |
| WB318 | ATGCAGGTACCTTACGCTTCTAATGTCTCTCGAATG |
| WB319 | ATGCAGCTAGCAGGAGGAAAGTGTTATATGTTTAAAGAAG | BCE_5606, NheI and KpnI sites are underlined |
| WB320 | ATGCAGGTACCGTCTACTCAACTAACATTAAGTAGAC |
| WB321 | ATGCAGCTAGCAAGAGGTGTTTTTAAATGATAGATT | BCE_5607, NheI and XbaI sites are underlined |
| WB322 | ATGCATCTAGACTCAATAGCTAATTCTTCTTTAAAC |
| WB323 | ATGCAGCTAGCAGGAGGGAGTGTGCAGAATGTTGCAAG | BCE_1018, NheI and KpnI sites are underlined |
| WB324 | ATGCAGGTACCCACTACCTCCCTAATATCTCTTAAC |
| WB391 | ATGCGAATTCATGACATTCTCAACCAACCAG | Nham_0569, EcoRI and KpnI sites are underlined |
| WB392 | GATCGGTACCTTATGCCGCAAGTCTCCGGGC |
| WB393 | ATGCGAATTCATGCACGGAATCGAAAAGCTCAACC | Nham_0582, EcoRI and KpnI sites are underlined |
| WB394 | GATCGGTACCCTACGTATGACTCAGCACATCGTCG |
| WB395 | AGCTGCTAGCAGGAGGTGTCGTATGCATAACATCCAATGC | Nham_0803, NheI and KpnI sites are underlined |
| WB396 | GATCGGTACCTCATTTGCCACCTCCATCGGTAG |
| WB397 | AGCTGCTAGCAGGAGGTGACCCATGCGCGATATCCAATGG | Nham_0842, NheI and KpnI sites are underlined |
| WB398 | GATCGGTACCTCATTTGCCACCTCCATCGGTAG |
| WB399 | ATGCGAATTCATGACGTCGCTGCCGCGTATTGG | Nham_1185, EcoRI and KpnI sites are underlined |
| WB400 | GATCGGTACCTTACGGCCAGGCTTTGGAGCGGC |
| WB401 | ATGCGAATTCATGGACGCTAAAGTCGTGAAGTCC | Nham_1353, EcoRI and KpnI sites are underlined |
| WB402 | GATCGGTACCCTATCCCAAGCGTTCGTTCCGGATC |
| WB403 | AGCTGCTAGCAGGAGGTGAGGTATGACCCCGGTTGTGATG | Nham_2515, NheI and KpnI sites are underlined |
| WB404 | GATCGGTACCTCACTCCGCTGCGACTTGAAATC |
| WB405 | ATGCGAATTCATGGGTGTGTCGCGTCGCGGG | Nham_3225, EcoRI and KpnI sites are underlined |
| WB406 | GATCGGTACCTCAGCCGGCCGCCATCTCGCT |
| WB407 | AGCTGCTAGCAGGAGGCTTTCATGAGCGAACGGGTCGAGCAGATCG | Nham_3845, NheI and KpnI sites are underlined |
| WB408 | GATCGGTACCTCACGTTTTTACAGGAGCATTATTGGCCC |
| WB409 | ATGCGAATTCATGAACGCCGTCGAGATTGAAG | Nham_4499, EcoRI and KpnI sites are underlined |
| WB410 | GATCGGTACCTCACGCGCTTACCTCCGTCTT |
|  |  |  |
| Primers for the *in vivo* assembly of MTase genes |  |  |
| WB325 | ATGCCATAGCATTTTTATCC | Amplification of BAMTA208_06525 from pWYE690 |
| WB475 | CGTAGTTTATTCATGAATTCCTCCTTCAACTATGTACTTGAGGTAATCGA |
| WB476 | TCGATTACCTCAAGTACATAGTTGAAGGAGGAATTCATGAATAAACTACG | Amplification of BAMTA208_6715 from pWYE691 |
| WB477 | TTATTGCTGTTCATGAATTCCTCCTTTATTCAGATTCTTTATTATCGTAT |
| WB478 | ATACGATAATAAAGAATCTGAATAAAGGAGGAATTCATGAACAGCAATAA | Amplification of BAMTA208_19835 from pWYE693 |
| WB479 | GAAAAAAAACGCATGAATTCCTCCTTTATTCTAAATCTAATAATTCATTT |
| WB480 | AAATGAATTATTAGATTTAGAATAAAGGAGGAATTCATGCGTTTTTTTTC | Amplification of BAMTA208_16660 from pWYE694 |
| WB326 | GATTTAATCTGTATCAGG |
| WB325 |  | Amplification of BCE_0393 from pWYE699 |
| WB575 | ATACAGTTCATATGTCTTACCTCCTTTAATCGGCGGTATTTTGTGTAGAT |
| WB576 | ATCTACACAAAATACCGCCGATTAAAGGAGGTAAGACATATGAACTGTAT | Amplification of BCE_4605 from pWYE700 |
| WB577 | TTTAAACATATAACACTTTCCTCCTTTACGCTTCTAATGTCTCTCGAATG |
| WB578 | CATTCGAGAGACATTAGAAGCGTAAAGGAGGAAAGTGTTATATGTTTAAA | Amplification of BCE_5606 from pWYE701 |
| WB579 | AATCTATCATTTAAAAACACCTCTTGTCTACTCAACTAACATTAAGTAGA |
| WB580 | TCTACTTAATGTTAGTTGAGTAGACAAGAGGTGTTTTTAAATGATAGATT | Amplification of BCE_5607 from pWYE702 |
| WB581 | ATATCATAATATCACTCTCCCTCCTCTCAATAGCTAATTCTTCTTTAAAC |
| WB582 | GTTTAAAGAAGAATTAGCTATTGAGAGGAGGGAGAGTGATATTATGATAT | Amplification of BCE_0365 from pWYE697 |
| WB583 | CTATCAACATACTTTTCCACCGCCTTCATTCTTTAATACTTGGCTCTACG |
| WB584 | CGTAGAGCCAAGTATTAAAGAATGAAGGCGGTGGAAAAGTATGTTGATAG | Amplification of BCE_0392 from pWYE698 |
| WB326 |  |
| WB325 |  | Amplification of Nham_0569 from pWYE712 |
| WB585 | TCGATTCCGTGCATGAATTCCTCCTTTATGCCGCAAGTCTCCGGGCGGCG |
| WB586 | CGCCGCCCGGAGACTTGCGGCATAAAGGAGGAATTCATGCACGGAATCGA | Amplification of Nham_0582 from pWYE713 |
| WB587 | GGATGTTATGCATACGACACCTCCTTCAGAGACTACGCACGTCGAGAATG |
| WB588 | CATTCTCGACGTGCGTAGTCTCTGAAGGAGGTGTCGTATGCATAACATCC | Amplification of Nham_0803 from pWYE714 |
| WB589 | CGCGACACACCCATGAATTCCTCCTTCATTTGCCACCTCCATCGGTAGAT |
| WB590 | ATCTACCGATGGAGGTGGCAAATGAAGGAGGAATTCATGGGTGTGTCGCG | Amplification of Nham_3225 from pWYE719 |
| WB326 |  |
|  |  |  |
| Primers for construction of pWYE561 |  |  |
| WB654 | TACGCGTCGACCGCTGATCACACGATAGTCGGCG | Amplification of P*Nham_3450* from the X14 strain, SalI site is underlined |
| WB655 | TCCTTTACTCATGATCCCTCGTCCTCAGATCCAT |
| WB656 | GGACGAGGGATCATGAGTAAAGGAGAAGAACTT | Amplification of *gfpmut3a* from pAD123, PstI site is underlined |
| WB650 | TGCAACTGCAGTTATTTGTATAGTTCATCCAT |
|  |  |  |
| Primers for construction of pWYE748 and *upp* inactivation |  |  |
| WB607 | CAAGGCGGACCGCTTATGCATG | Amplification of *upp* upstream homologous sequences |
| WB608 | CTTTAGTTGAAGCAAATACGTAAACCTTTCCCAT |
| WB609 | TTTACGTATTTGCTTCAACTAAAGCACCCATTAGTTC | Amplification of chloramphenicol resistance gene from pMK4 |
| WB610 | AGTCTGTCACCCAACCTTCTTCAACTAACGGGGCAGG |
| WB611 | TTGAAGAAGGTTGGGTGACAGACTGTACGGAAC | Amplification of *upp* downstream homologous sequences |
| WB612 | TCCCGAGTGATCGTATGGAC |
| WB605 | AACACTTCGCGGACCGCGCG | Primers flanking the homologous sequences for detection purpose |
| WB606 | TGCCACACTGACTTTGTCGG |
