# Supplementary material for: A Mimicking-of-DNA-Methylation-Patterns Pipeline for Overcoming the Restriction Barrier of Bacteria
Source: PLoS Genet. 2012 Sep 27;8(9):e1002987. doi: 10.1371/journal.pgen.1002987 (PMC3459991; doi:10.1371/journal.pgen.1002987)
Supplement: Text S1 — Supporting Information Methods and Supporting Information Results. The construction and validation of E. coli EC135 is described in detail. (DOC) [file pgen.1002987.s012.doc]

**Text S1. Supporting Information**

**Supporting Information Methods**

**Gene inactivation and site-directed mutagenesis in *E. coli* TOP10**

The *dam* and *dcm* genes were inactivated using the lambda RED mediated method [1]. Functional restoration of *recA1* was achieved by replacing it with a wild-type allele from *E. coli* W3110 using the temperature sensitive plasmid pKOV [2]. Restoration of *recA* was tested by DNA sequencing and resistance to nalidixic acid (NA) [3]. The EC067 and EC132 strains were cultured to OD_600_ of 0.5 in LB medium; NA was added to a final concentration of 100 μg/mL, and then samples were drawn in 30 min intervals and washed with 0.9% NaCl. Aliquots were spread on LB plates and cultured overnight at 37°C. Survival rates were calculated using the colonies at time 0 as a control. The Dam inactivated strain was verified for its sensitivity to 2-aminopurine (2-AP). Bacterial cultures (5 μL) at 10^5^, 10^6^ and 10^7^ CFU/mL were spotted on LB plates containing 100 μg/mL 2-AP and cultured overnight at 37°C. The absence of methylated bases in GAmTC and CCmWGG sequences of the mutated strains were also tested by restriction digestion. Chromosomal DNA (500 ng) from the mutated and reference strains was digested with Sau3AI, DpnI and DpnII to test for the loss of Dam function and BstNI and PspGI for the loss of Dcm function (New England Biolabs).

**Supporting Information Results**

**Verification of *dcm* and *dam* deletion and *recA* site-directed mutagenesis in *E. coli***

The *dcm* gene was deleted in the TOP10 strain to yield strain EC067. Successful deletion of *dcm* was verified by PCR and sequencing (Figure S1A). Dcm modifies the internal cytosine of the CCmWGG sequence, thereby conferring resistance to PspG1 to the chromosomal DNA of the TOP10 strain. PspG1 cleavage is blocked by methylation in the recognition sequence, but the sequence is still sensitive to BstNI digestion, which cleaves both methylated and unmethylated DNA. Nevertheless, DNA prepared from the EC067, EC132 and EC135 strains are sensitive to both BstNI and PspGI digestion as shown in Figure S1B.

The *recA1* gene of the EC067 strain was mutated to the wild type allele to obtain the EC132 strain. Site-directed mutagenesis of *recA* was verified by DNA sequencing. Functional restoration of the *recA+* mutant was determined by its resistance to nalidixic acid (NA), which induces the SOS response in *recA*+ bacteria but selectively, and reversibly, blocks DNA replication in susceptible bacteria [3]. As shown in Figure S1C, the EC067 strain is more sensitive than the *recA+* mutant EC132 strain to NA.

The *dam* gene of the EC132 strain was deleted to create the EC135 strain. Deletion of *dam* was verified by PCR and sequencing (Figure S1D). D*am* mutation was also validated by digesting the chromosomal DNA with REases that recognize the same GATC sequence but have different methylation preferences (Figure S1E). Chromosomal DNA from the EC135 strain was readily digested by DpnII, which prefers unmethylated DNA, but not by DpnI, which digests only methylated DNA. It has been reported that undirected mismatch repair initiated by the incorporation of the base analog 2-aminopurine (2-AP) kills DNA methylation-deficient *dam* mutants by inducing DNA double-strand breaks [4]. This method was used to validate the loss of Dam function in this study. When compared with the parental strains, drops of EC135 cell cultures at 10^5^ and 10^6^ CFU/mL only grew to discrete colonies on LB plates containing 2-AP (Figure S1F).

**Supporting Information References**

1. Datsenko KA, Wanner BL (2000) One-step inactivation of chromosomal genes in *Escherichia coli* K-12 using PCR products. Proc Natl Acad Sci U S A 97: 6640-6645.

2. Link AJ, Phillips D, Church GM (1997) Methods for generating precise deletions and insertions in the genome of wild-type *Escherichia coli*: application to open reading frame characterization. J Bacteriol 179: 6228-6237.

3. Newmark KG, O'Reilly EK, Pohlhaus JR, Kreuzer KN (2005) Genetic analysis of the requirements for SOS induction by nalidixic acid in *Escherichia coli*. Gene 356: 69-76.

4. Matic I, Ekiert D, Radman M, Kohiyama M (2006) Generation of DNA-free *Escherichia coli* cells by 2-aminopurine requires mismatch repair and nonmethylated DNA. J Bacteriol 188: 339-342.
